# Exosomal LINC00460/miR-503-5p/ANLN positive feedback loop aggravates pancreatic cancer progression through regulating T cell–mediated cytotoxicity and PD-1 checkpoint

**DOI:** 10.1186/s12935-022-02741-5

**Published:** 2022-12-08

**Authors:** Jun Yao, Ruoyu Gao, Minghan Luo, Defeng Li, Liliangzi Guo, Zichao Yu, Feng Xiong, Cheng Wei, Benhua Wu, Zhenglei Xu, Dingguo Zhang, Jianyao Wang, Lisheng Wang

**Affiliations:** 1 Department of Gastroenterology, Jinan University of Second Clinical Medical Sciences, Shenzhen Municipal People’s Hospital, No. 1017, East Gate Road, Shenzhen City, 518020 Guangdong Province China; 2 Department of General Surgery, Shenzhen Children’s Hospital, No. 7019, Yitian Road Road, Shenzhen City, 518026 Guangdong Province China

**Keywords:** Pancreatic cancer, LINC00460, miR-503-5p, ANLN, Exosomes, M2 polarization

## Abstract

**Background:**

Long non-coding RNA (lncRNA) LINC00460 is an onco-lncRNA in a variety of cancers, including pancreatic cancer (PC). This study is aimed to investigate the regulatory mechanisms of LINC00460 in PC.

**Methods:**

The tumor and adjacent normal tissues were collected from 73 PC patients. The expression of LINC00460, miR-503-5p, and ANLN was detected using qRT-PCR. We then analyzed the proliferation, migration, invasion, and apoptosis/cell cycle of PC cells by performing the MTT/EdU, transwell, and flow cytometry assays, respectively. The xenograft tumor model were utilized to confirm the effect of LINC00460 knockdown on PC through anti-PD-1 therapy in vivo, and the sensitivity of PANC-1 cells to the cytotoxicity of CD8^+^ T cells in vitro. Western blotting was used to determine the protein levels. A co-culture model was utilized to explore the effects of exosomes on macrophages.

**Results:**

LINC00460 was up-regulated in PC tissues and cells. LINC00460 knockdown suppressed cell proliferation, migration, and invasion, facilitated cell apoptosis and G0/G1 phase arrest, and inhibited the tumor growth through anti-PD-1 therapy. Both miR-503-5p down-regulation and ANLN up-regulation reversed the effects of LINC00460 knockdown on inhibiting the proliferation, migration and invasion, and on promoting the apoptosis, G0/G1 phase arrest, and the sensitivity of PC cells to the cytotoxicity of CD8^+^ T cells. Exosomes were uptaken by the ambient PC cells. PANC-1 cells-derived exosomal LINC00460-induced M2 macrophage polarization accelerates the cell migration and invasion.

**Conclusions:**

LINC00460 silencing attenuates the development of PC by regulating the miR-503-5p/ANLN axis and exosomal LINC00460-induced M2 macrophage polarization accelerates the migration and invasion of PANC-1 cells, thus LINC00460 may act as a possible therapeutic target for treating PC.

**Supplementary Information:**

The online version contains supplementary material available at 10.1186/s12935-022-02741-5.

## Background

Pancreatic cancer (PC) is a type of malignant tumor with poor prognosis in global counties, typically contributing to high mortality [1, 2]. Until now, surgery, chemotherapy, and radiotherapy are the leading therapeutic strategies for PC, among which surgery is considered as the only way to cure PC [3]. Some emerging strategies also show great therapeutic potential in PC, such as targeted therapy, immunotherapy, and microbial therapy [4]. Because of no obvious symptoms during PC progression, PC is usually diagnosed at an advanced stage with the presence of metastasis [5]. Unfortunately, the 5-year survival rate of patients at an advanced stage is only 2%-9% [3]. Therefore, understanding the regulatory mechanisms that underlie PC is critical for developing a new treatment strategy. Additionally, it is well known exosomes are a kind of round membranous vesicles existed in extracellular microenvironment [6–8]. Previous studies have been revealed that exosomes play crucial roles in the tumorigenesis of most types of cancers, such as lung, prostate, pancreas, breast, ovarian, and thyroid cancers [9, 10]. In consequence, to explore the detailed effects of exosomes on PC progression is of great significance in clinic.

More and more attentions have been paid to long non-coding RNAs (lncRNAs) due to dysregulated expression of lncRNAs is participated in modulating tumorigenesis and progression of PC [11–14]. Numerous studies have reported some lncRNAs serve as suppressors in PC progression, such as LINC-PINT (pancreatic ductal adenocarcinoma) [15], LINC00261 (PC) [11] and LINC01963 (PC) [12]. In addition, other researchers have confirmed that lncRNAs may also have promoting effects on the tumorigenesis of PC [13, 14, 16]. For instance, LINC01420 acts as an onco-lncRNA in human PC cell lines and xenograft tumor model in mice, thus accelerating the development of PC [13]. LINC00152 facilitates the proliferation, migration and invasion of human PC cells to aggravate PC [14]. Similarly, based on in vitro experiments in human PC cells, LINC-RoR is also determined to play a morbigenous role in PC tumorigenesis [16]. More importantly, a recent study conducted by Sun et al. has revealed that LINC00460 expression may be correlated with the survival rate of PC patients [17]. Nevertheless, the accurate function and detailed regulatory mechanism of LINC00460 in PC progression need to be further explored.

Additionally, immunotherapy has been identified as a crucial therapeutic approach in cancers. Activation of the immune system can reduce their immune escape [18]. The most common immunotherapy is the interaction of programmed death ligand 1 (PD-L1) with its receptor programmed death-1 (PD-1) in tumor-specific T cells [19, 20]. PD-L1 can interact with PD-1 to reduce the activity of CD8^+^ T cells leading to the immune evasion in tumors [21–23]. Therefore, blocking PD-1/PD-L1 checkpoint is considered as an efficient tool for the immunotherapy in tumors [24, 25], also including PC [26, 27]. The roles of lncRNAs in immune cells have recently been reported, particularly in cancer immunotherapy. For instance, in ovarian cancer, lncRNA HOTTIP potentiates immune escape through inducing PD-L1 signaling in neutrophils and enhancing production of IL-6 [28]. In granulocytic myeloid-derived suppressor cells, lncRNA PVT1 can modulate immunosuppression activity [29]. LncRNA FENDRR can sponge miR-423-5p to reduce immune escape in hepatocellular carcinoma [30]. Furthermore, natural killer (NK) cell and cytotoxic T lymphocyte (CTL) also have crucial roles in anti-cancer immunity and suppression of cancer related inflammation [31, 32]. In particular, CTL are a critical component of the anti-tumour immune response, as they lyse tumour cells and suppress tumour metastasis [32]. In this study, we further concentrated on the function of LINC00460 in the cytotoxic activity of T cells and anti-PD-1 therapy toward PC cells.

In recent years, increasing evidences have indicated the anti-tumor roles of microRNAs (miRNAs) in PC [33–35]. Luo et al. have demonstrated that overexpression of miR-33b reverses the promoting effects of lncRNA DNACR on the proliferation and metastasis of PC cells [33]. Liu et al. have discovered that significant reductions of proliferation and migration were observed in PC cells after transfection of miR-28-5p mimics [34]. Zhu et al. have confirmed that the abilities of proliferation, migration and invasion in PC cells were restrained by miR-139-5p, eventually attenuating the tumor progression of PC [35]. Interestingly, as a member of miRNAs, miR-503 is also identified to be an anti-tumor miRNA in human cancers, such as non-small-cell lung cancer (NSCLC) [36], cervical cancer (CC) [37–39] and breast cancer (BC) [40]. At the same time, miR-503 is involved in pancreas development of mouse [41] and overexpression of miR-503 accelerates the programmed apoptosis of human pancreatic cells [42]. However, the possible role of miR-503-5p in PC progression, and whether LINC00460 regulates miR-503-5p on PC remain unclear.

Anillin (ANLN), as an oncogene in several types of human cancers, has a positive effect on tumorigenesis [43–46]. UP-regulation of ANLN is observed in various human malignancies and exhibits a poor prognosis of cancers, such as in nasopharyngeal carcinoma (NPC) [43], lung cancer [44], colorectal cancer (CRC) [47], cervical cancer [48] and breast cancer [45, 49]. Notably, researches conducted by Olakowski et al., Guo et al. and Idichi et al. have all demonstrated that the overexpressed ANLN dramatically expedites the advancement of PC [46, 50, 51]. But there are few studies on ANLN interaction with miR-503 involving in the PC progression.

In this study, the influence of LINC00460 silencing on the proliferation, migration, invasion, apoptosis, and cell cycle of PC cells, the potential regulatory mechanisms of LINC00460/miR-503-5p/ANLN on PC progression, as well as the effects of exosomal LINC00460 on macrophage polarization and metastasis of PC cells were investigated. The results of our study may develop a new treatment strategy for PC.

## Methods

### Samples collection

Totally 73 PC patients diagnosed by pathological examinations were selected in our hospital from 2016 to 2018. All these patients had not received any treatments for PC before admission. The tumor and adjacent normal tissues from patients were collected by surgery and histologically confirmed. Each patient in this study obtained the written informed consent. The protocols of this study were approved by ethical committee in Shenzhen Municipal People’s Hospital (No. 2018194).

### Cell culture, grouping and transfection

Five human PC cell lines (ASPC-1, BxPC-3, SW1990, PANC-1 and Mia-PaCa-2) and human monocytic cells THP-1 were procured from Procell Life Science & Technology, Ltd (Wuhan, China). Human normal pancreatic ductal epithelial cell line (H6C7) was procured from Chuanqiu Biotech, Ltd (Shanghai, China). At the conditions of 37˚C with 5% CO_2_, all the above cells were cultured in RPMI-1640 medium (#11,875,085, Thermo Fisher Scientific, Carlsbad, CA, USA) containing 10% fetal bovine serum (FBS; #10,100,147, Thermo Fisher Scientific).

ShRNA-LINC00460-1/-2 (sh-LINC00460-1/-2) and their negative control (sh-NC) were procured from Generalbiol, Ltd (Chuzhou, China). Overexpression-ANLN (pcDNA-ANLN), Overexpression-LINC00460 (pcDNA-LINC00460) and its negative control (pcDNA-NC), miR-503-5p mimics, miR-503-5p inhibitor and their negative control (miR-NC) were all procured from Ribo Biotech, Ltd (Guangzhou, China). The aforementioned agents were transfected into SW1990 and PANC-1 cells using a Lipofectamine RNAiMAX kit (#13,778,150, Invitrogen, Carlsbad, CA, USA). After treatment for 48 h, the transfected cells were harvested to perform the following trails. Additionally, THP-1 cells were utilized to incubate with PMA (100 ng/mL) for 24 h to reduce differentiation into macrophages.

### Quantitative reverse-transcription PCR (qRT-PCR)

Total RNA was extracted from PC tissues and cell lines (ASPC-1, BxPC-3, SW1990, PANC-1 and Mia-PaCa-2) using a TRIzol Plus RNA kit (#12,183,555, Invitrogen) and reversely transcribed into cDNA using the GoScript reverse transcription system (#A5001, Promega, Madison, WI, USA). Then the cDNA was subjected to qRT-PCR analysis. The thermocycling conditions were as follows: 94˚C for 5 min, followed by 40 cycles at 94˚C for 10 s, 60˚C for 40 s and 72˚C for 1 min. GADPH and U6 were used as the internal references. Gene expression was quantified using the 2^−ΔΔCt^ method and normalized to a control group.

### MTT assay

The viability of PC cells was detected by MTT assay. In brief, the transfected PC cell lines (PANC-1 and SW1990 cells) were seeded into a 96-well plate with 2 × 10^5^ cells per well. Subsequently, the cells were incubated for 24, 48, 72 and 96 h, followed by adding 20 µl MTT (#C11019, Ribobio) to each well at 37˚C. After incubation for another 2 h, the viability (OD450) was determined by a microplate reader (Thermo Fisher Scientific).

### EdU proliferation assay

The proliferation of PC cell lines (PANC-1 and SW1990 cells) was assessed using an EdU assay kit (#C10310, Ribobio) based on the manufacturer’s instructions. Briefly, the cells were seeded into a 24-well plate at a density of 1 × 10^5^ cells per well, cultured with 50 μM EdU for 2 h at 37 °C, followed by fixing with 4% formaldehyde, permeabilizing using 0.5% Triton X-100 for 20 min and incubating with 1 × Apollo reaction cocktail for 30 min at room temperature. After that, DAPI (4',6-diamidino2-phenylindole) was utilized to stain DNA for another 30 min. The EdU-positive cells were observed under a fluorescence microscope (Carl Zeiss, Oberkochen, Germany).

### Flow cytometry analysis

The apoptosis and cell cycle of PC cells was evaluated using the Annexin V- FITC apoptosis detection kit (#331,200, Thermo Fisher Scientific) and cell cycle kit (#A10798, Thermo Fisher Scientific) in accordance with the manufacturers’ protocol, respectively. For detecting apoptosis, cells were re-suspended in binding buffer and stained with Annexin V-EGFP and PI at 4 °C for 15 min in the dark. For detecting cell cycle, cells were fixed in 70% ethanol and incubated with cell cycle reagent at 4˚C for 30 min in the dark. Both cell apoptosis and cell cycle were assessed using a FACScan flow cytometer (Becton, Dickinson and Company, Franklin Lakes, NJ, USA).

### Transwell assay for migration and invasion

For migration assay, PANC-1 and SW1990 cells (5 × 10^4^) were re-suspended in a serum-free medium and seeded into the upper chamber. At the same time, the lower chamber was added with RPMI-1640 medium containing 10% FBS. For the invasion assay, Matrigel was used to extra coat the membranes before adding the cells. Following incubation for overnight at 37 °C C, cells in the lower chamber were stained with 0.1% crystal violet for 15 min. Light microscope (magnification, × 400) was used to count the stained cells in five randomly-selected fields.

### Tumor xenografts in nude mice

This study was performed in the Animal Experimental Center of our hospital and approved by the ethical committee in Shenzhen Municipal People’s Hospital (No. 20180224001). Healthy BALB/c nude mice (weighing 20 ± 2 g) were procured from Vital River Laboratories (Beijing, China) and fed under a 12 h cycle (12 h for light and 12 h for dark) environment. The mice were divided into the sh-NC group and the sh-LINC00460-1 group (each group for 5 mice). The sh-LINC00460-1 or sh-NC was firstly integrated into the lentiviral vector, followed by transfecting into PANC-1 cells. Next, the transfected PANC-1 cells (1 × 10^5^ cells/100 ul) were subcutaneously injected into the right flanks of the nude mice. During duration of this study, we measured tumor volumes every other week. After 4 weeks, mice were anesthetized with pentobarbital sodium (50 mg/kg), followed by sacrificing using cervical dislocation method. The tumor xenograft was separated from mice and weighted.

### Mice xenografts anti-PD-1 therapy assay

The healthy BALB/c mice (weighing 20 ± 2 g) were initially divided into the sh-NC group and the sh-LINC00460-1 group (each group for 10 mice). Similar to the above experimental procedures, the transfected PANC-1 cells (1 × 10^5^ cells/100 ul) were subcutaneously injected into the right flanks of the mice. Afterwards, the mice were randomly assigned to 4 groups: the IgG + sh-NC, anti-PD-1 + sh-NC, IgG + sh-LINC00460-1, and anti-PD-1 + sh-LINC00460-1 groups (each group for 5 mice). Then, the mice were injected in the tail vein with anti-PD-L1 antibody (200 μg/mouse) or IgG (200 μg/mouse) three times each week for 2 weeks. Tumor weight was measured after mice were killed.

### Tumor-specific cytotoxicity by T cells

T cells were plated in 96-well plates (1 × 10^6^ cells/well). The transfected PANC-1 cells were incubated with CD8^+^ T cells at a ratio of 1:2.5, 1:5, and 1:10 (PANC-1:CD8^+^) for 24 h. Lactic dehydrogenase (LDH) in supernatants was then detected using a CytoTox 96^®^ Non-Radioactive Cytotoxicity Assay (Promega) according to the manufacturer’s protocol. The absorbance at 450 nm for each reaction was measured. Percent cytotoxicity was calculated as follows: Cytotoxicity (%) = (Expeimental—Effector Spontaneous—Target Spontaneous)/(Target Maximum—Target Spontaneous) × 100%.

### Immunohistochemistry (IHC)

IHC staining was conducted using streptavidin–biotin-peroxidase complex method. Briefly, pancreatic cancer tissue samples were fixed, paraffin-embedded, dewaxed, rehydrated, and antigen retrieval. Then samples were stained with primary antibody Ki67 (1: 1,500; #ab15580, Abcam, Cambridge, MA, USA) at 4˚C overnight, followed by incubation with HRP-conjugated secondary antibody (1:3,000; #ab288151, Abcam) for 30 min at 37 °C. Pictures were taken under a light microscope (magnifications, × 400).

### RNA-binding protein immunoprecipitation (RIP) assay

RIP assay was performed using MagnaRIP RNA-Binding Protein Immunoprecipitation Kit (#17–704, Millipore, Bedford, MA, USA). In brief, the pcDNA-MS2/pcDNA-MS2-LINC00460 and pBobi-MS2-GFP obtained from OBiO Technology (Shanghai, China) were co-transfected into the SW1990 and PANC-1 cells for 48 h. Subsequently, the cells were lysed with RIP lysis buffer. The extracts of cells were incubated with RIP buffer magnetic beads with anti-GFP or Mice IgG (#ab13970, Abcam) at 4 ˚C for overnight and then washed by RIP wash buffer. After that, the eluates were collected and we detected the expression of miRNAs (miR-491-5p, miR-503-5p, miR-654-3p, miR-320a and miR-320b) using qRT-PCR.

### Dual-luciferase reporter gene (DLR) assay

The predicted binding region sequences were inserted into pGL3 to establish the wild-type. For constructing the mutant-type, the mutation sequences were inserted into pGL3. SW1990 and PANC-1 cells were then co-transfected with LINC00460/ANLN-Mut or LINC00460/ANLN-WT and miR-503-5p mimics/miR-NC at 37 °C. After 48 h of culture, a dual-luciferase reporter assay system (#E1910, Promega) was used to detect the luciferase activity.

### Isolation of exosomes

After transfection, PANC-1 cells were sequentially cultured in RPMI-1640 medium containing 10% FBS without exosomes at 37 °C with 5% CO2. After 72 h of culture, the supernate and cell fragments were separated using centrifuges. The collecting supernatant was utilized to extract the exosomes using a GM™ Exosome Isolation Reagent kit (E3002, Geneseed Biology, Guangzhou, China) and the mixtures were incubated at 4 °C for 30 min. Subsequently, the mixtures were centrifuged at 2000 g for 30 min. The exosomes were observed under a transmission electron microscopy (TEM) and identified using the surface markers of exosomes TSG101, CD63 and CD81 by western blot.

### Western blot analysis

RIPA buffer containing protease inhibitors was used to extract proteins from cells, followed by detecting the protein concentrations using the BCA Protein Assay Kit (#ab102536, Abcam). Approximately 30 µg proteins were separated by 10% sodium dodecyl sulphate polyacrylamide gel electrophoresis (SDS-PAGE) and transferred into polyvinylidene fluoride (PVDF) membrane. Membrane blocking was performed using 5% bovine serum albumin (BSA) at room temperature. Next, the membrane was incubated overnight at 4˚C with primary antibodies against E-cadherin (1:1,500; #ab40772, Abcam), N-cadherin (1:1,500; #ab18203, Abcam), ANLN (1:1,500; #ab211872, Abcam), TSG101 (1:1,500; #ab133586, Abcam), CD63 (1:1,500; #ab134045, Abcam) and CD81 (1:1,500; #ab109201, Abcam), β-actin (1:1,500; #ab8227, Abcam). Then, tris-buffered saline Tween-20 (TBST) was used to wash the membranes for 3 times. Subsequently, at room temperature, the HRP-conjugated IgG secondary antibody (1:3,000; #sc2357, Santa Cruz, Waltham, MA, USA) was added to incubate for 1 h. β-actin served as the internal reference. The membrane was developed by Chemiluminescence reagents (#WP20005, Thermo Fisher Scientific) under a Gel-Pro analyzer (version 4.0, USA).

### Fluorescence-labeled exosomes and uptake of the exosomes assay

Based on the manufacturer's instructions, the Exo-Green fluorescent staining kit (#PKH67, Yanzai Biology, Shanghai, China) was used to label the exosomes. In brief, the exosomes were re-suspended with PBS and mixed with Exo-Green to incubate for 10 min. After the mixture was centrifuged, the precipitates (containing exosomes) were re-suspended with PBS. Cells (1 × 10^5^) were seeded into a 35-mm dish. Subsequently, the labeled exosomes (100 μl) were added, cultured for 24 h, and observed under a confocal microscope (magnification, × 400).

### Establishment of cell co-culture model

PANC-1 cells (without transfection) were seeded into the upper chamber of Transwell. After 24 h, macrophages transfected with exosomes (exo)-pcDNA-LINC00460/exo-pcDNA-NC or without transfection were seeded into the lower chamber of transwell culture plate. All the aforementioned cells were seeded with 2 × 10^5^ cells per well. Following co-culture for 24 h, the abilities migration and invasion in PANC-1 cells were detected.

### Statistical analysis

SPSS Statistics software (version 20.0, USA) was used to perform statistical analyses. Data were presented as the means ± standard deviation (SD). Student *t*-tests were used to assess the differences between two groups, whereas one-way ANOVA followed by Tukey's multiple comparisons test was used to evaluate the differences among multiple groups. Pearson’s correlation analysis was used to determine the correlations among LINC00460, miR-503-5p and ANLN in PC tissues. Survival curves were calculated by the Kaplan-Meier method and compared using the log-rank test. P-value less than 0.05 indicated a statistically significant difference. All experiments were conducted in triplicate in at least three independent trials.

## Results

### LncRNA LINC00460 expression is up-regulated in PC tissues and PC cells

**Fig. 1:**
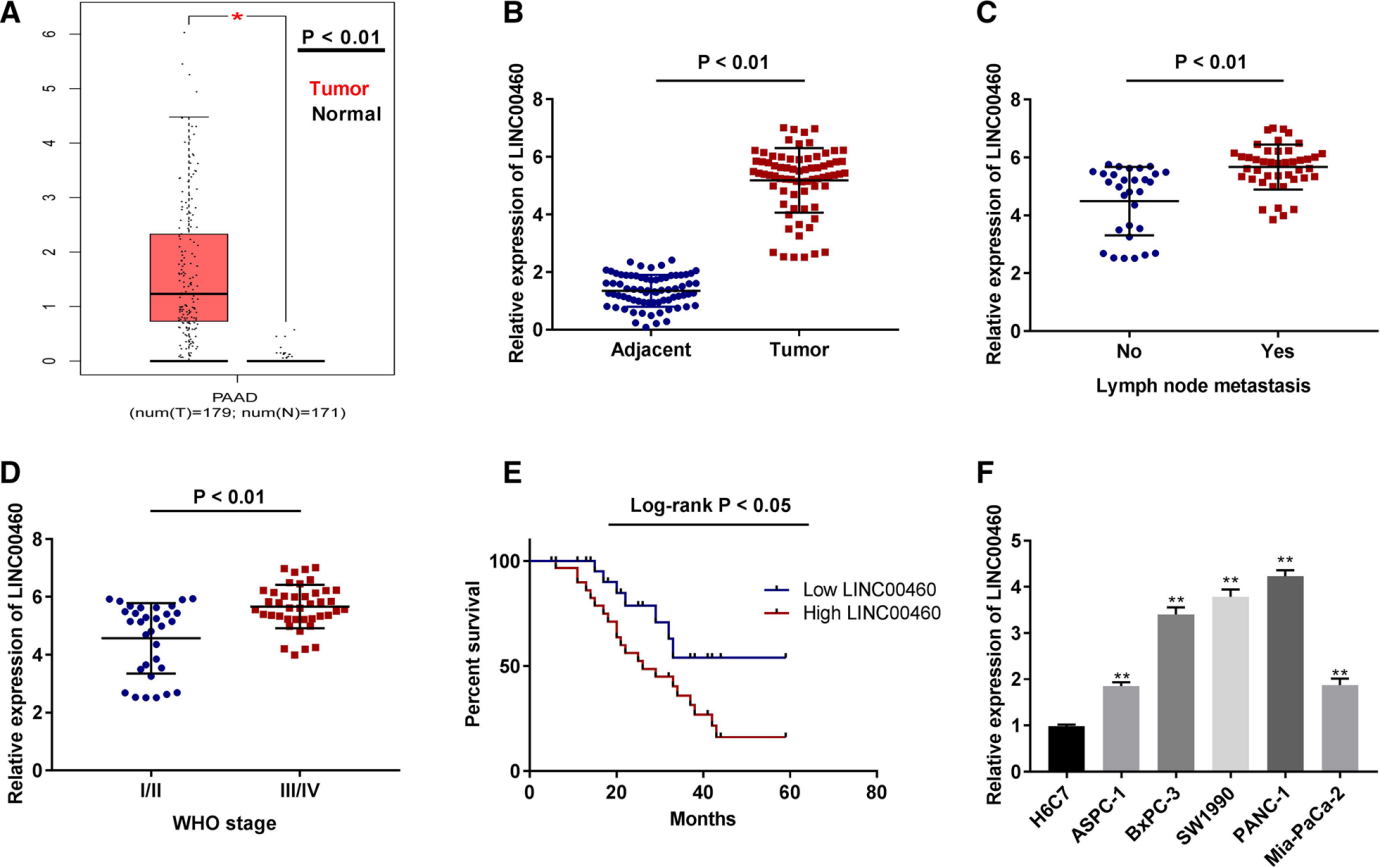
LINC00460 is highly expressed in PC tissues and cells. **A** The expression of LINC00460 in PAAD tissues and normal tissues was analyzed in TCGA. *P < 0.01. **B** The expression of LINC00460 in PC tissues (n = 73) and adjacent tissues (n = 73) was detected by qRT-PCR. *P* < 0.01. **C** The expression of LINC00460 in lymph node metastasis or non-metastasis was detected by qRT-PCR. *P* < 0.01. **D** The expression of LINC00460 at different WHO grades was detected by qRT-PCR. *P* < 0.01. **E** Correlation between LINC00460 expression and overall survival of PC patients was assessed using Kaplan–Meier analysis. *P < 0.05. **F** The expression of LINC00460 in H6C7 cells and PC cell lines (ASPC-1, BxPC-3, SW1990, PANC-1 and Mia-PaCa-2) was detected by qRT-PCR. **P < 0.01 *vs*. the H6C7 cells group

Based on TCGA database, LINC00460 expression in pancreatic adenocarcinoma (PAAD) and normal tissues was analyzed. We found that the expression of LINC00460 was elevated in PAAD tissues compared to that in normal tissues (Fig. 1A, P < 0.01). In addition, the results of qRT-PCR displayed that in comparison to the adjacent tissues, LINC00460 expression was significantly up-regulated in tumor tissues (Fig. 1B, P < 0.01), and was also higher in lymph node metastasis of PC patients than that of non-metastasis patients (Fig. 1C, P < 0.01). At the same time, an increased expression of LINC00460 was detected in stage III/IV of PC compared to that in stage I/II (Fig. 1D, P < 0.01). According to the median of LINC00460 expression in tumor tissues, low and high expression groups were categorized. As presented in Fig. 1E and Table 1, high expression of LINC00460 was strongly correlated with worse survival rate (Fig. 1E, P < 0.05), metastasis (*P* = 0.013) and World Health Organization (WHO) Grade (*P* = 0.047) (Table 1). Meanwhile, LINC00460 expression in PC cell lines (ASPC-1, BxPC-3, SW1990, PANC-1 and Mia-PaCa-2) was dramatically increased in contrast to that in H6C7 cells (Fig. 1F, P < 0.01). The expression of LINC00460 in SW1990 and PANC-1 cell lines was higher than that in other cell lines. Therefore, SW1990 and PANC-1 cell lines were chosen to perform the following experiments.

**Table 1:** Correlations between lncRNA LINC00460 expression and clinicopathological characteristics in PC

Characteristics	Total	LINC00460 expression		*P*-value
		Low(36)	High(37)	
Age				0.719
< 60	39	20	19	
≥ 60	34	16	18	
Gender				0.733
Male	40	19	21	
Female	33	17	16	
Diameter				0.291
< 2 cm	45	20	25	
≥ 2 cm	28	16	12	
Metastasis				0.013*
NO	30	20	10	
YES	43	16	27	
WHO Grade				0.047*
I/II	32	20	12	
III/IV	41	16	25	

^*^P < 0.05, WHO, World Health Organization

### *LncRNA LINC00460 knockdown inhibits the malignant characteristics of PC cells *in vitro*, and suppressed the growth of tumor xenograft *in vivo

**Fig. 2:**
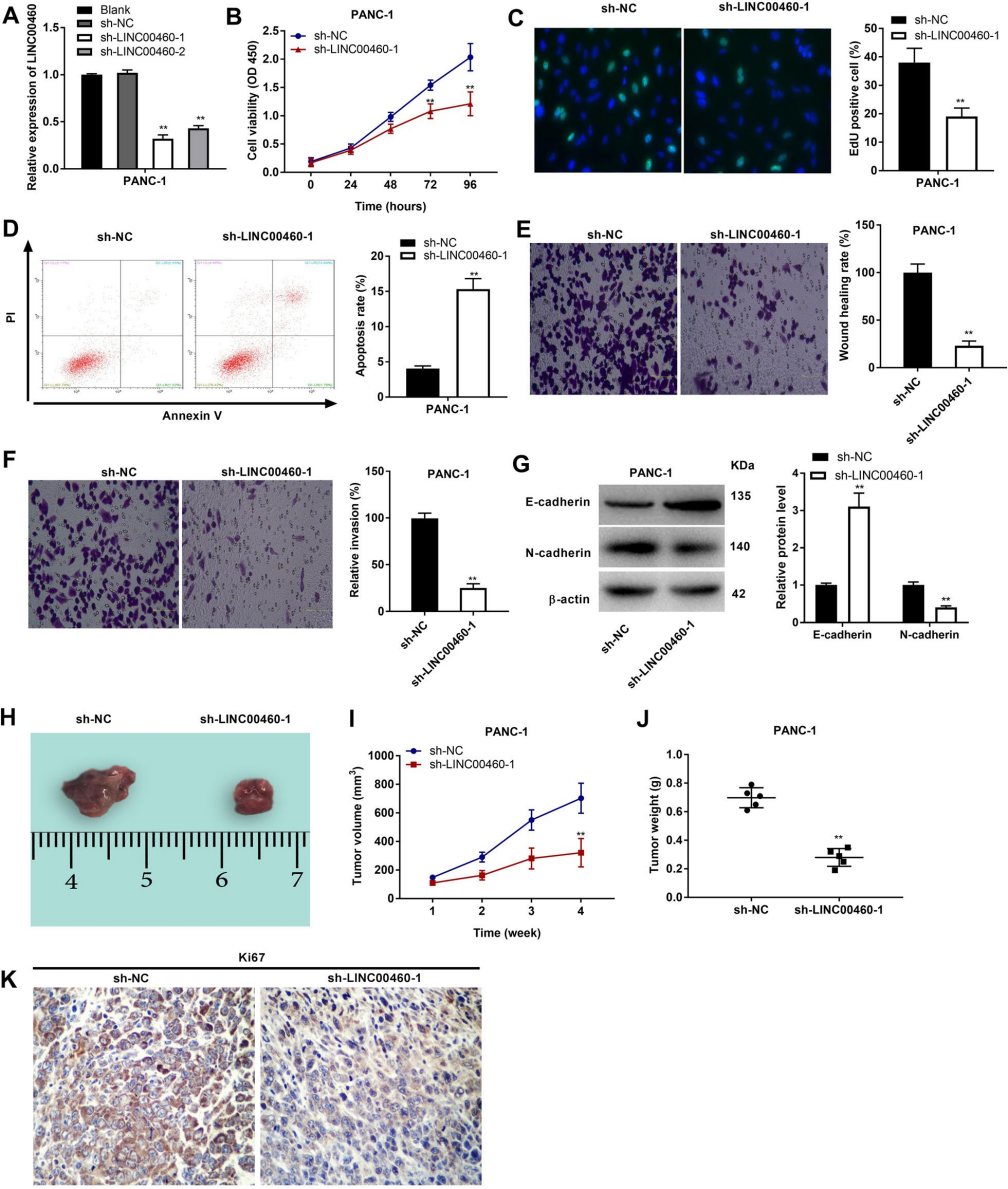
LINC00460 knockdown inhibits the malignant characteristics of PANC-1 cells in vitro and suppresses the growth of tumor xenograft in vivo. **A** The expression of LINC00460 in PANC-1 cells was detected by qRT-PCR. **B** The viability (OD450) of PANC-1 cells was measured by MTT assay. **C** The proliferation of PANC-1 cells was determined by EdU assay (200 ×). **D** The apoptosis of PANC-1 cells was analyzed by flow cytometry assay. (E) The migration ability of PANC-1 cells was measured by transwell assay. **F** The invasion ability of PANC-1 cells was measured by transwell assay. **G** The protein levels of E-cadherin and N-cadherin were determined by Western blot. **H** The images of tumor xenograft with LINC00460 knockdown. **I** The tumor volumes were monitored at different time points. **J** The tumor weights were measured 4 weeks later. (K) Ki67 staining in PC tissues was measured by IHC assay (400 ×). **P < 0.01 *vs*. the sh-NC group

sh-LINC00460-1/-2 was transfected into PANC-1 cells to determine the transfection efficiency. As expected, LINC00460 expression was reduced after transfection (Fig. 2A, P < 0.01). Because the transfection efficiency of sh-LINC00460-1 was relatively high, it was chosen to perform the subsequent experiments. MTT assay showed the decreased cell viability in the sh-LINC00460-1 group compared to the sh-NC group (Fig. 2B, P < 0.01). EdU assay further verified that cell proliferation was also dampened by LINC00460 knockdown (Fig. 2C, P < 0.01). In addition, the transfection of sh-LINC00460-1 into PANC-1 cells significantly accelerated cells apoptosis (Fig. 2D, P < 0.01). The migration ability and invasion ability of PANC-1 cells were both suppressed by transfection of sh-LINC00460-1 (Fig. 2E, F, P < 0.01). Furthermore, through western blot analysis, we discovered the protein level of E-cadherin (an epithelial-mesenchymal transition marker) was elevated, whereas the protein level of N-cadherin (another marker) was inhibited by transfection of sh-LINC00460-1 (Fig. 2G, P < 0.01). Consistent results on sh-LINC00460-1 were also revealed in SW1990 cells (Additional file 1: Fig S1A-G, *P* < 0.01).

The effect of LINC00460 knockdown on the growth of tumor xenograft was also explored. We discovered that injection of sh-LINC00460-1 into mice dramatically inhibited the growth of tumor xenograft (including tumor volume and tumor weight) (Fig. 2H–J, P < 0.01). As shown in Fig. 2K, Ki67 staining was also visibly alleviated by injection of sh-LINC00460-1 (*P* < 0.01).

### MiR-503-3p is identified to be a target of LINC00460

**Fig. 3:**
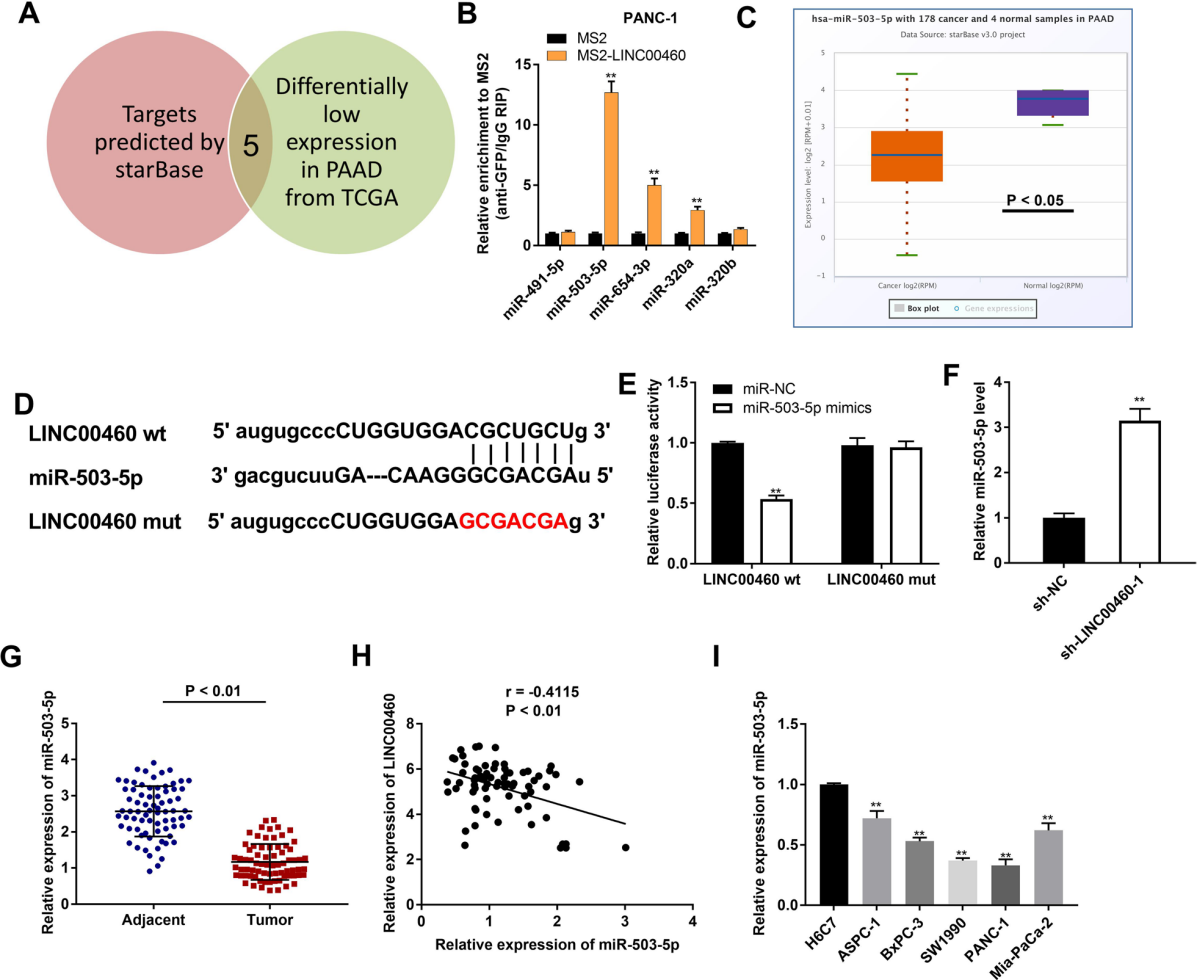
MiR-503-5p is the direct target of LINC00460. **A** The predicted targets of LINC00460 from databases (StarBase and TCGA) were exhibited by Venn diagram. **B** Five miRNAs selected from databases were detected by RIP assay in PANC-1 cells. **P < 0.01 *vs*. the MS2 group. **C** The expression of miR-503-5p in PAAD tissues and normal tissues was analyzed in TCGA. *P < 0.05. **D** The predicted binding site between LINC00460 and miR-503-5p. **E** The luciferase activity in PANC-1 cells transfected with pGL3-LINC00460 WT/pGL3-LINC00460 MUT and miR-503-5p mimics/NC was determined by DLR assay. **P < 0.01 *vs*. the miR-NC group. **F** The expression of miR-503-5p after transfection of sh-LINC00460-1/NC into PANC-1 cells was detected by qRT-PCR. **P < 0.01 *vs*. the sh-NC group. **G** The expression of miR-503-5p in tumor tissues (n = 73) and adjacent tissues (n = 73) was detected by qRT-PCR. *P* < 0.01. **H** The correlation between LINC00460 and miR-503-5p. *P* < 0.01, r = −0.4115. **I** The expression of miR-503-5p in H6C7 cells and PC cell lines (ASPC-1, BxPC-3, SW1990, PANC-1 and Mia-PaCa-2) was detected by qRT-PCR. **P < 0.01 *vs*. the H6C7 cells group

We discovered five potential miRNAs were modulated by LINC00460 in accordance with Venn diagram (Fig. 3A). Next, these five miRNAs were utilized to screen out the target of LINC00460 by RIP assay. The results disclosed that the LINC00460 immunoprecipitates obtained from PC cell lines (SW1990 and PANC-1) were notably enriched with miR-503-5p, miR-654-3p and miR-320a compared to those in the empty vector (MS2) group (Fig. 3B and Additional file 2: Fig. S2A, *P* < 0.01). MiR-503-5p was chosen to further verify whether it is the target of LINC00460 due to its highest expression among these three miRNAs. We used the Starbase software to predict the potential binding site between LINC00460 and miR-503-5p (Fig. 3D). Subsequently, DLR assay was performed in PC cell lines to further determine the relationship between LINC00460 and miR-503-5p. The results presented that the luciferase activity in the LINC00460-WT/miR-503-5p mimics group was obviously decreased in contrast to that in LINC00460-WT/miR-NC group (Fig. 3E and Additional file 2: Fig. S2B, *P* < 0.01). After transfection of sh-LINC00460-1 into PC cell lines, an increased expression of miR-503-5p was detected by qRT-PCR (Fig. 3F and Additional file 2: Fig. S2C, *P* < 0.01). Interestingly, TCGA database illustrated a relative low expression of miR-503-5p in PAAD tissues by contrast to normal tissues (Fig. 3C, P < 0.05). Similarly, the results of qRT-PCR demonstrated that miR-503 was minimally expressed not only in tumor tissues (Fig. 3G, P < 0.01) but also in PC cell lines (ASPC-1, BxPC-3, SW1990, PANC-1 and Mia-PaCa-2) (Fig. 3I, P < 0.01). Besides, there was a significant inverse correlation between LINC00460 and miR-503-5p in PC tissues (Fig. 3H; *P* < 0.01, r = −0.415).

### Overexpression of miR-503-5p suppresses the malignant characteristics of PC cells

**Fig. 4:**
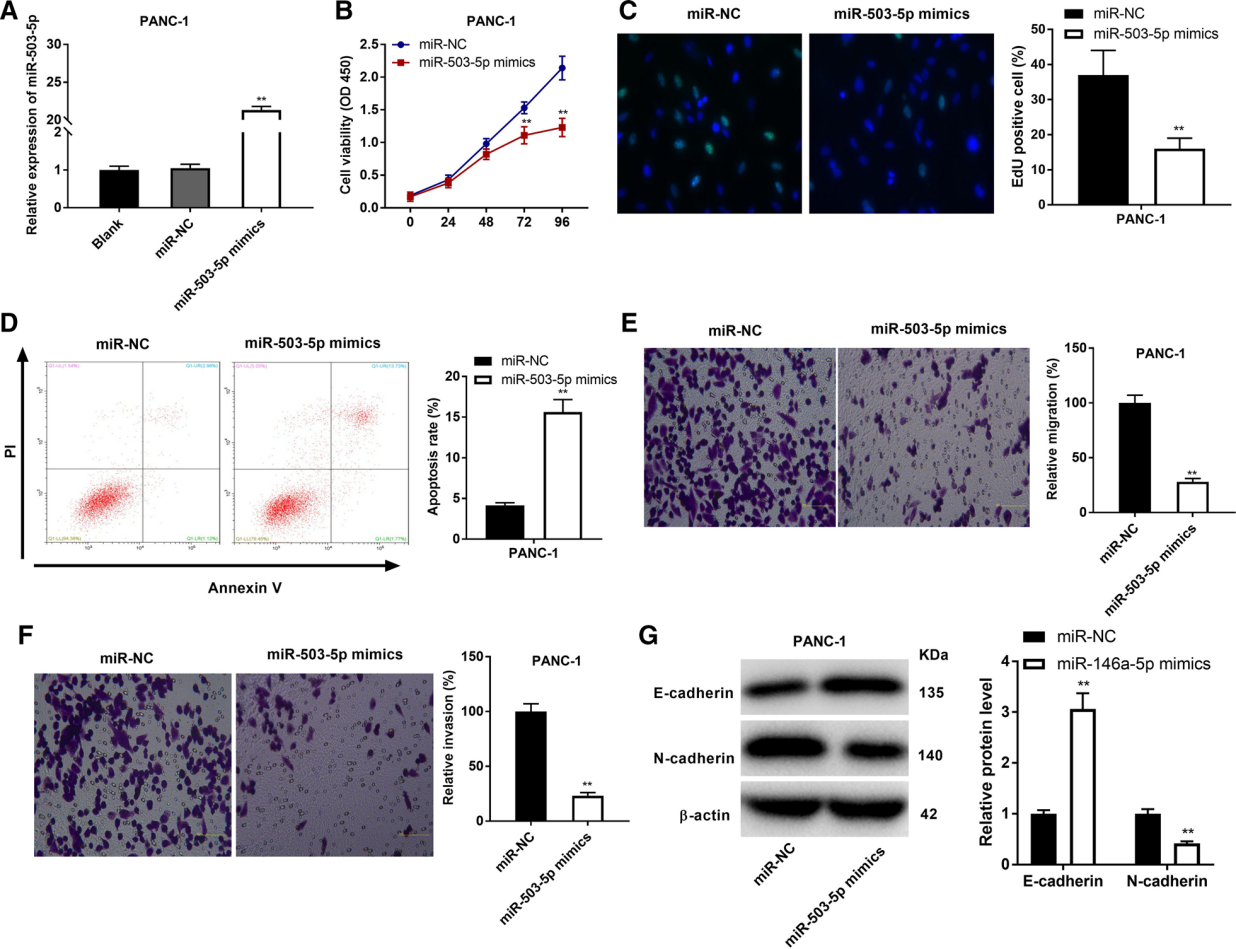
Overexpression of miR-503-5p inhibits the malignant characteristics of PANC-1 cells. **A** The expression of miR-503-5p after transfection of miR-503-5p mimics/NC into PANC-1 cells was detected by qRT-PCR. **B** The viability (OD450) of PANC-1 cells was measured by MTT assay. **C** The proliferation of PANC-1 cells was determined by EdU assay (200 ×). **D** The apoptosis of PANC-1 cells was analyzed by flow cytometry assay. **E** The migration ability of PANC-1 cells was measured by transwell assay. **F** The invasion ability of PANC-1 cells was measured by transwell assay. **G** The protein levels of E-cadherin and N-cadherin were determined by Western blot. **P < 0.01 *vs*. the miR-NC group

To study the role of miR-503-5p on the biological processes of PC cells, miR-503-5p mimics or miR-NC was transfected into PANC-1 cells (Fig. 4A, P < 0.01). As shown in Fig. 4B–F, up-regulation of miR-503-5p suppressed cell proliferation, migration, and invasion, and accelerated cell apoptosis (*P* < 0.01). Western blot assay further indicated that the level of E-cadherin was promoted, while N-cadherin was inhibited by miR-503-5p overexpression (Fig. 4G). Consistently, miR-503-5p overexpression also suppressed the malignant characteristics of SW1990 cells (Additional file 3: Fig. S3A-G, *P* < 0.01).

### ANLN is a target gene of miR-503-5p

**Fig. 5:**
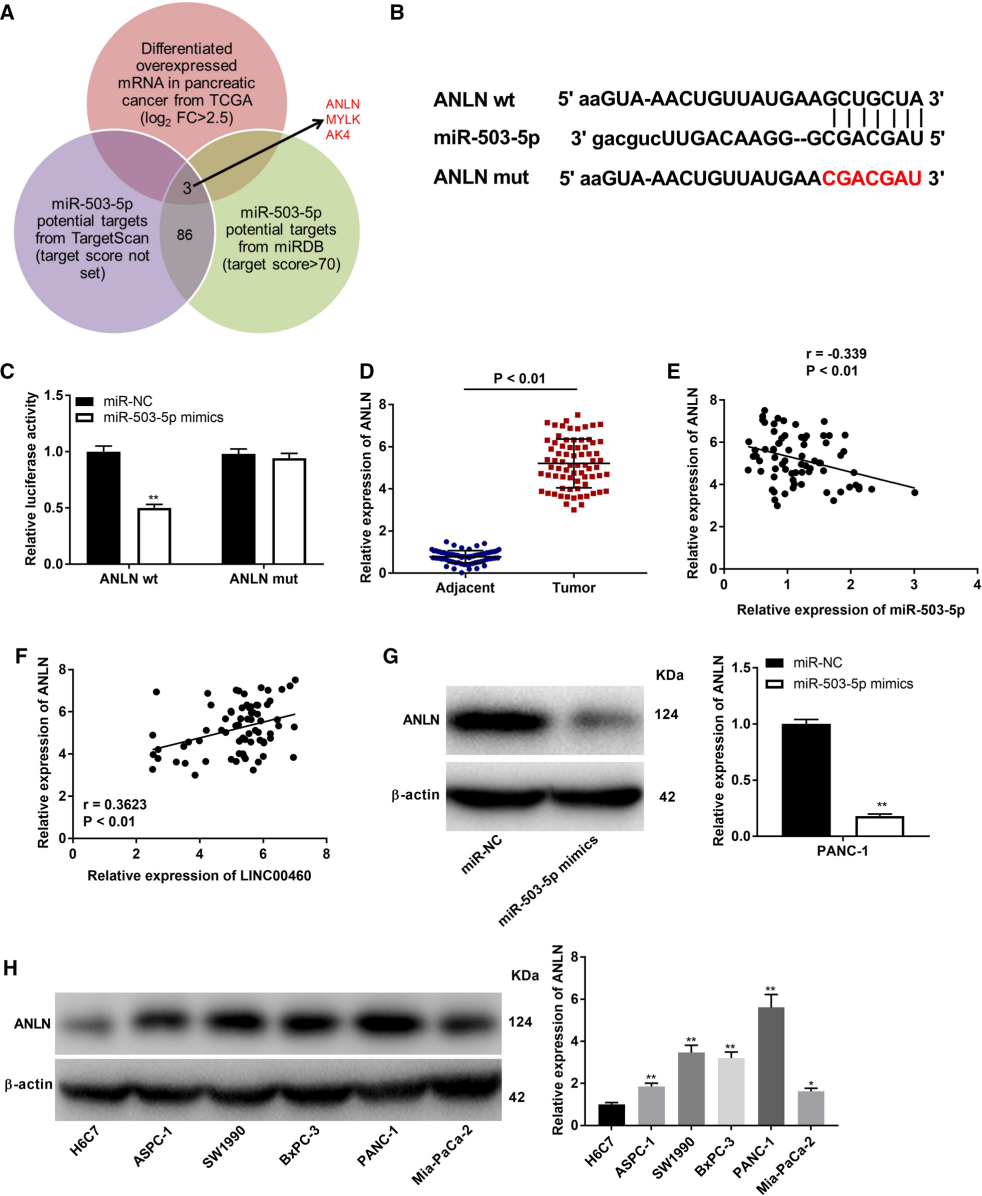
Identification of ANLN as the target gene of miR-503-5p. **A** Three target genes of miR-503-5p from databases (TargetScan, miRDB and TCGA) were exhibited by Venn diagram. **B** The predicted complementary binding site of miR-503-5p and ANLN. **C** The luciferase activity in PANC-1 cells transfected with pGL3-ANLN WT/pGL3-ANLN MUT and miR-503-5p mimics/NC was determined by DLR assay. **P < 0.01 *vs*. the miR-NC group. **D** The expression of miR-503-5p in tumor tissues (n = 73) and adjacent tissues (n = 73) was detected by qRT-PCR. *P* < 0.01. **E** The correlation between miR-503-5p and ANLN. *P* < 0.01, r = −0.339. **F** The correlation between LINC00460 and ANLN. *P* < 0.01, r = −0.3623. **G** The protein level of ANLN after transfection of miR-503-5p mimics/NC into PANC-1 cells was determined by western blot assay. **P < 0.01 *vs*. the miR-NC group. **H** The protein level of ANLN in H6C7 cells and PC cell lines (ASPC-1, BxPC-3, SW1990, PANC-1 and Mia-PaCa-2) were determined by western blot assay. **P < 0.01 *vs*. the H6C7 cells group

**Fig. 6:**
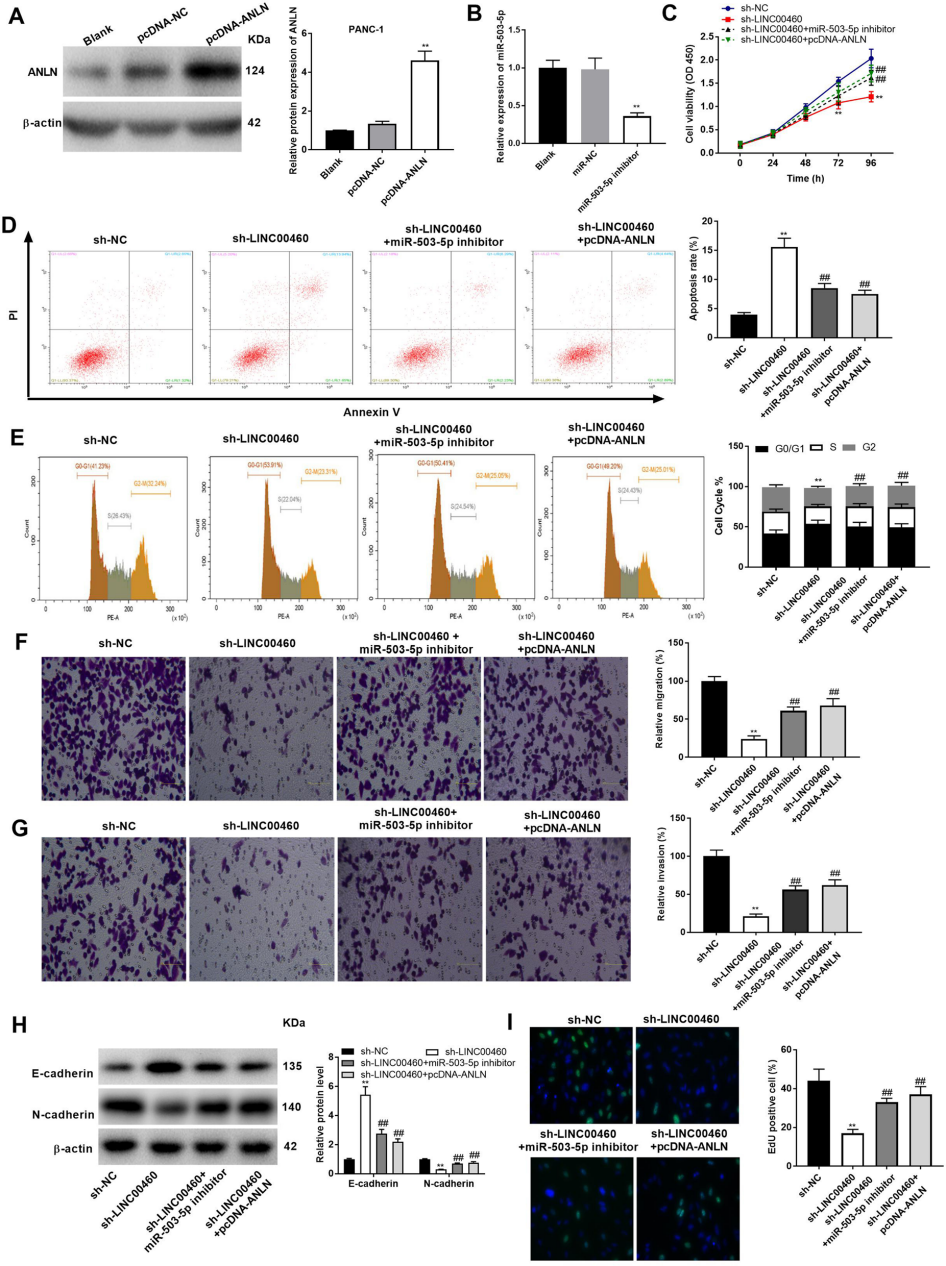
LINC00460 knockdown inhibits the proliferation, migration and invasion, and promotes the apoptosis and G0/G1 phase arrest of PANC-1 cells by regulating miR-503-5p/ANLN. **A** The protein level of ANLN after transfection of pcDNA-ANLN/NC into PANC-1 cells was measured by western blot assay*.* **P < 0.01 *vs*. the pcDNA-NC group. **B** The expression of miR-503-5p after transfection of miR-503-5p inhibitor/miR-NC into PANC-1 cells was measured by qRT-PCR. **P < 0.01 *vs*. the miR-NC group. **C** The viability (OD450) of PANC-1 cells was measured by MTT assay. **D** The apoptosis of PANC-1 cells was analyzed by flow cytometry assay. **E** The cell cycle of PANC-1 cells was analyzed by flow cytometry assay. **F** The migration ability of PANC-1 cells was measured by transwell assay. **G** The invasion ability of P PANC-1 cells was measured by transwell assay. **H** The protein levels of E-cadherin and N-cadherin were determined by Western blot. **I** The proliferation of PANC-1 cells was determined by EdU assay (200 ×). **P < 0.01 *vs*. the sh-NC group, ^##^*P* < 0.01 *vs*. the sh-LINC00460 group

With the prediction of TCGA database, TargetScan, and miRDB in Venn diagram, three target genes (ANLN, MYLK and AK4) of miR-503-5p were found (Fig. 5A). Among these three genes, ANLN is selected as the research target in following assays due to its important role in PC. Through TargetScan software, the underlying binding site between miR-503-5p and ANLN was shown in Fig. 5B. MiR-503-5p mimics could visibly decrease the luciferase activity of ANLN WT reporter vector in both SW1990 and PANC-1 cells (Fig. 5C and Additional file 4: Fig. S4A, *P* < 0.01). The results of qRT-PCR uncovered that ANLN expression was higher in tumor tissues than that in adjacent tissues (Fig. 5D, P < 0.01). Similar to the above data, relative high level of ANLN protein was determined by western blot assay in PC cell lines (ASPC-1, BxPC-3, SWw1990, PANC-1 and Mia-PaCa-2) (Fig. 5H, P < 0.01). Additionally, ANLN expression was negatively correlated with miR-503-5p (Fig. 5E; *P* < 0.01, r = −0.339) and was positively correlated with LINC00460 (Fig. 6F; *P* < 0.01, r = 0.3623). Furthermore, we displayed that transfection of miR-503-5p mimics into SW1990 and PANC-1 cells dramatically reduced the protein level of ANLN (Fig. 5G and Additional file 4: Fig. S4B, *P* < 0.01).

### *Silencing of LINC00460 attenuates the progression of PC through modulating the miR-503-5p/ANLN axis *in vitro

To explore the regulatory mechanisms among LINC00460, miR-503-5p and ANLN on PC progression, feedback verification experiments were performed in PANC-1 cells. First, we detected the transfection efficiency of pcDNA-ANLN and miR-503-5p inhibitor. Western blot uncovered that pcDNA-ANLN distinctly elevated the level of ANLN protein (Fig. 6A, P < 0.01), and qRT-PCR showed that miR-503-5p inhibitor decreased the expression of miR-503-5p in PANC-1 cells (Fig. 6B, P < 0.01). As expected, both down-regulation of miR-503-5p and up-regulation of ANLN reversed the effect of LINC00460 silencing on promoting cell apoptosis, migration, and invasion, as well as on inhibiting cell proliferation in PANC-1 cells (Fig. 6C, D, F, G, and I, P < 0.01). Cell cycle assay determined that the transfection of sh-LINC00460 increased G0/G1 phase cells and decreased G2 phase cells (*P* < 0.05), and these effects were weakened by miR-503-5p silencing or ANLN overexpression (Fig. 6E, P < 0.01). In addition, miR-503-5p silencing and ANLN overexpression also reversed the effects of sh-LINC00460 on up-regulating E-cadherin and down-regulating N-cadherin (Fig. 6H, P < 0.01).

### *Silencing of LINC00460 reduces the tumor growth of PC through anti-PD-1 therapy and enhances the sensitivity of PANC-1 cells to cytotoxicity of CD8*^+^*T cells *via* regulation of the miR-503-5p/ANLN axis*

**Fig. 7:**
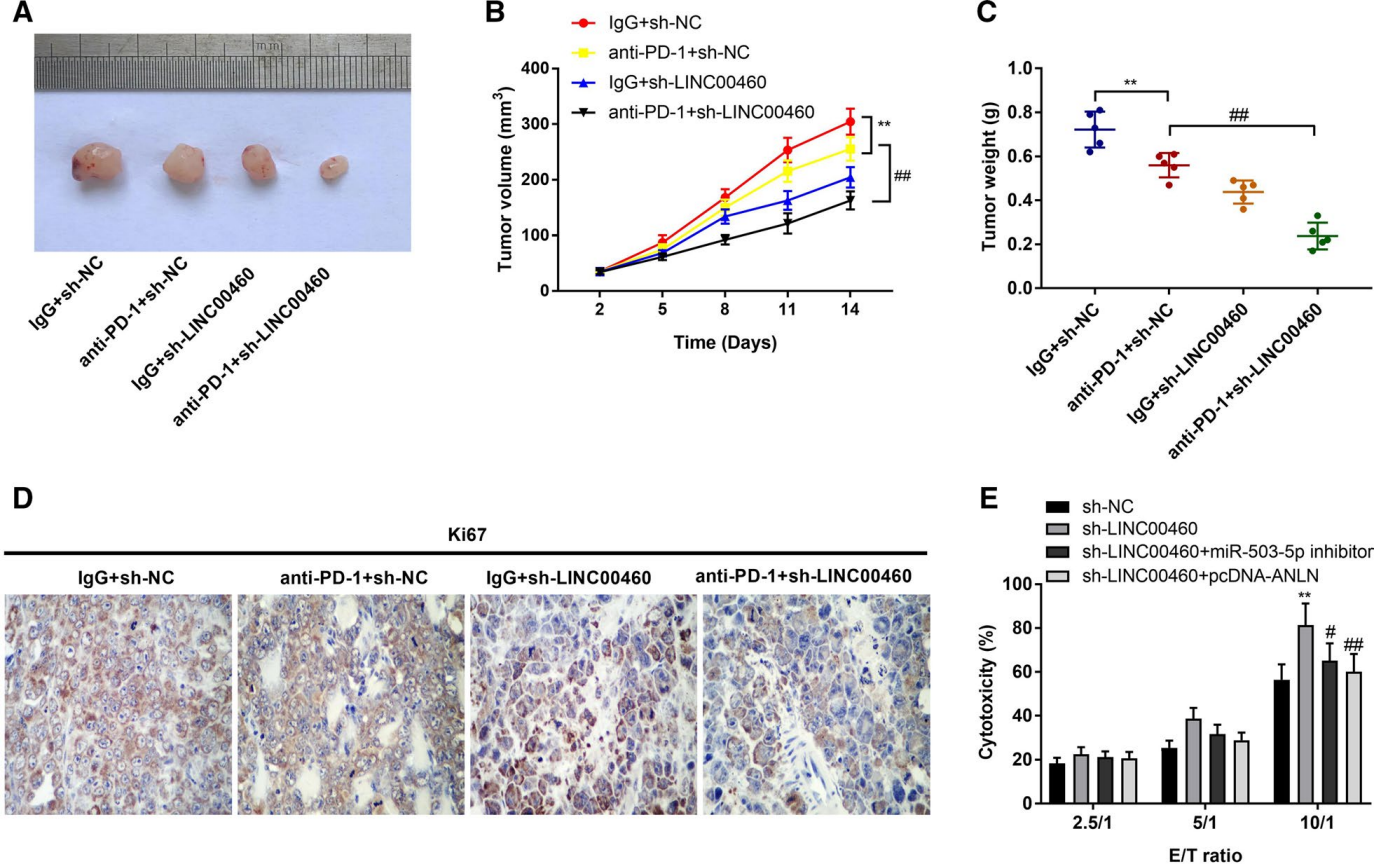
Silencing of LINC00460 reduces the tumor growth of PC through anti-PD-1 therapy and enhances the sensitivity of PANC-1 cells to cytotoxicity of tumor-reactive T cells via regulating the miR-503-5p/ANLN axis. **A** The images of tumor xenograft with different treatments. **B** The tumor volumes were monitored at different time points. **C** The tumor weight in mice of each group after 2 weeks of injection. **P < 0.01 *vs*. the IgG + sh-NC group; ^##^*P* < 0.01 *vs*. the anti-PD-1 + sh-NC group. **D** Ki67 staining in PC tissues was measured by IHC assay (400 ×). **E** The sensitivity of PANC-1 cells to the cytotoxicity of tumor-reactive T cells in different treatments. **P < 0.01 *vs*. the sh-NC group; ^#^*P* < 0.05, ^##^*P* < 0.01 *vs*. the sh-LINC00460 group

To further evaluate the effect of sh-LINC00460-1 on anti-PD-1 therapy in vivo, the transfected cells (sh-NC or sh-LINC00460-1) were injected into mice treated with or without anti-PD-1 to establish the xenografts. We observed that the use of anti-PD-1 in mice led to the generation of smaller tumors compared to those use of IgG (*P* < 0.01). Interestingly, the application of sh-LINC00460-1 enhanced the inhibitory effect of anti-PD-1 therapy on tumor growth in mice (Fig. 7A–C, P < 0.01). Meanwhile, Ki67 IHC staining showed that the xenograft derived from the anti-PD-1 + sh-NC group had less proliferative cells compared to the IgG + sh-NC group. The proliferative cells in the anti-PD-1 + sh-LINC00460-1 group were further less compared to those of the anti-PD-1 + sh-NC group (Fig. 7D). Next, the tumor-killing abilities of T cells from the mice in different groups were assessed. As illustrated in Fig. 7E, the cytotoxicity of CD8^+^T cells from mice treated with sh-LINC00460-1 to PANC-1 cells (10: 1) was higher than those treated with sh-NC (*P* < 0.01). However, the relatively high cytotoxicity of CD8^+^ T cells to PANC-1 cells caused by sh-LINC00460-1 were reversed by the low expression of miR-503-5p (*P* < 0.05) or high expression of ANLN (*P* < 0.01).

### Exosomal-LINC00460 facilitates M2 macrophage polarization

**Fig. 8:**
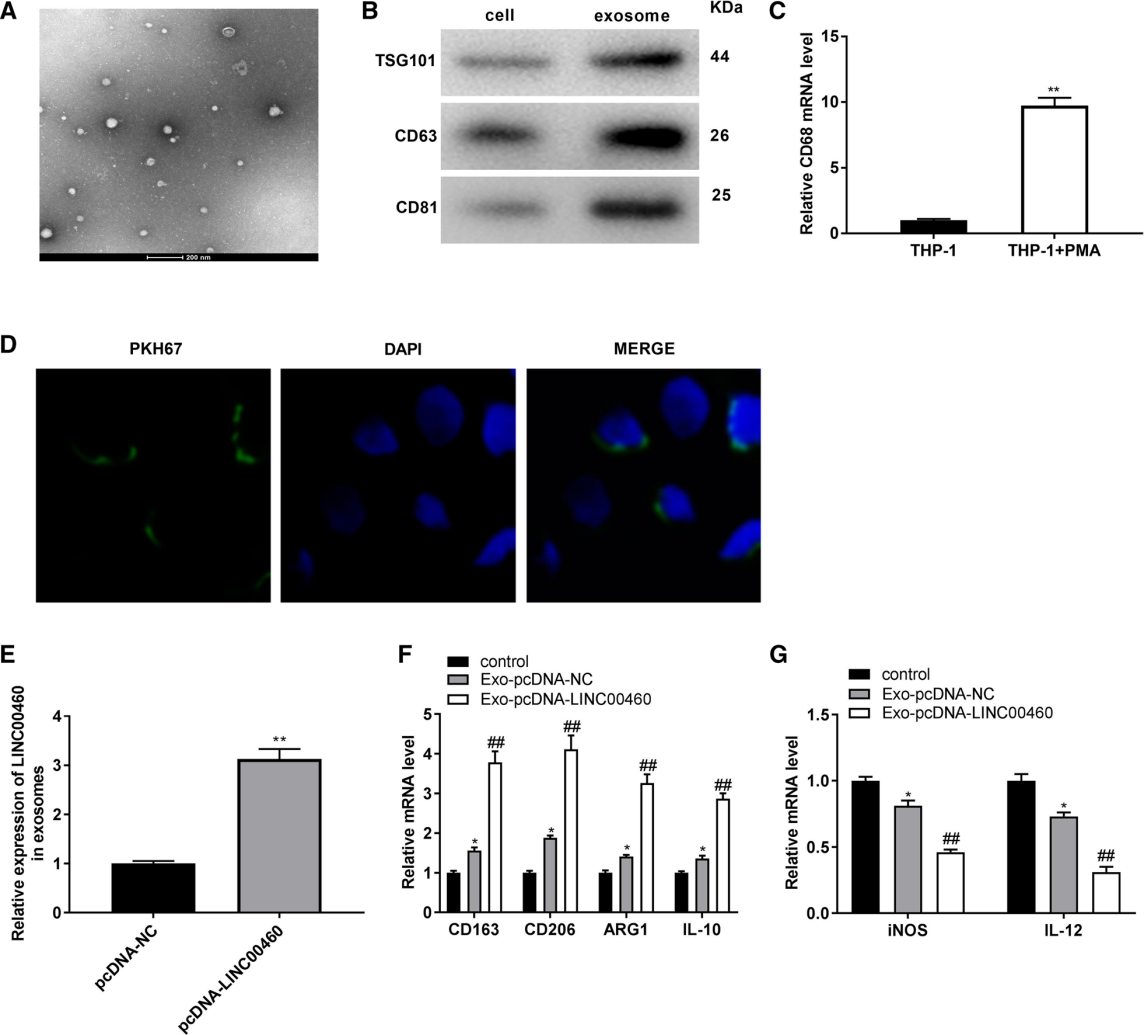
Exosomal-LINC00460 facilitates M2 macrophage polarization. **A** The morphological characteristics of exosomes which was round membranous vesicle were observed by a TEM. Scale bar: 200 nm. **B** The levels of exosomes surface marker (TSG101, CD63 and CD81) were detected by Western blot. **C** The mRNA level of macrophage marker CD68 was detected by qRT-PCR. **P < 0.01 *vs*. the THP-1 cells group. **D** Uptake of exosomes was observed by a confocal microscope (400 ×). **E** The expression of LINC00460 in exosomes secreted by PANC-1 cells was detected by qRT-PCR. **P < 0.01 *vs*. the pcDNA-NC group. **F** The mRNA levels of M2 polarized markers (CD163, CD206, ARG1 and IL-10) were detected by qRT-PCR. *P < 0.01 *vs*. the control group; ^##^*P* < 0.01 *vs*. the Exo-pcDNA-NC group. (G) The mRNA levels of M1 polarized markers (iNOS and IL-12) were detected by qRT-PCR. *P < 0.01 *vs*. the control group; ^##^*P* < 0.01 *vs*. the Exo-pcDNA-NC group

Under a TEM, the white precipitates isolated from PANC-1 cells were some round membranous vesicles (30–60 nm), which was in accordance with the characteristics of exosomes (Fig. 8A). Subsequently, the exosomes were authenticated by western blot assay. We disclosed that the expression levels of exosomal markers (TSG101, CD63 and CD81) in the exosomes group were increased obviously compared to those in whole cells group (Fig. 8B). Therefore, we confirmed that the exosomes were extracted successfully. Next, we detected the mRNA level of CD68 (macrophage marker) by qRT-PCR. The results indicated that CD68 mRNA level was significantly increased in the THP-1 + PMA group in contrast to the THP-1 group (Fig. 8C, P < 0.01), suggesting that THP-1 cells was differentiated into macrophages successfully. After that, the fluorescence-labeled exosomes were observed using a confocal microscope, and we found that the exosomes were uptaken by macrophages and intensively distributed around the cytoplasm (Fig. 8D). After transfection of pcDNA-LINC00460 into PANC-1 cells, we detected the expression of LINC00460 in the exosomes secreted by PANC-1 cells. The results of qRT-PCR indicated that pcDNA-LINC00460 dramatically up-regulated LINC00460 expression in exosomes (Fig. 8E, P < 0.01). Interestingly, compared with the controls group, the increased mRNA levels of M2 polarization markers (CD163, CD206, ARG1 and IL-10) (Fig. 8F, P < 0.05) and decreased mRNA levels of M1 polarization markers (iNOS and IL-12) (Fig. 8G, P < 0.05) were exhibited in the exo-pcDNA-NC groups. Whereas in comparison to the exo-pcDNA-NC groups, exo-pcDNA-LINC00460 further elevated the mRNA levels of M2 polarization markers (Fig. 8F, P < 0.01) and inhibited the mRNA levels of M1 polarization markers (Fig. 8F, P < 0.01), suggesting that exosomal LINC00460 facilitates M2 macrophage polarization.

### M2 macrophage polarization aggravates the migration and invasion of PANC-1 cells

**Fig. 9:**
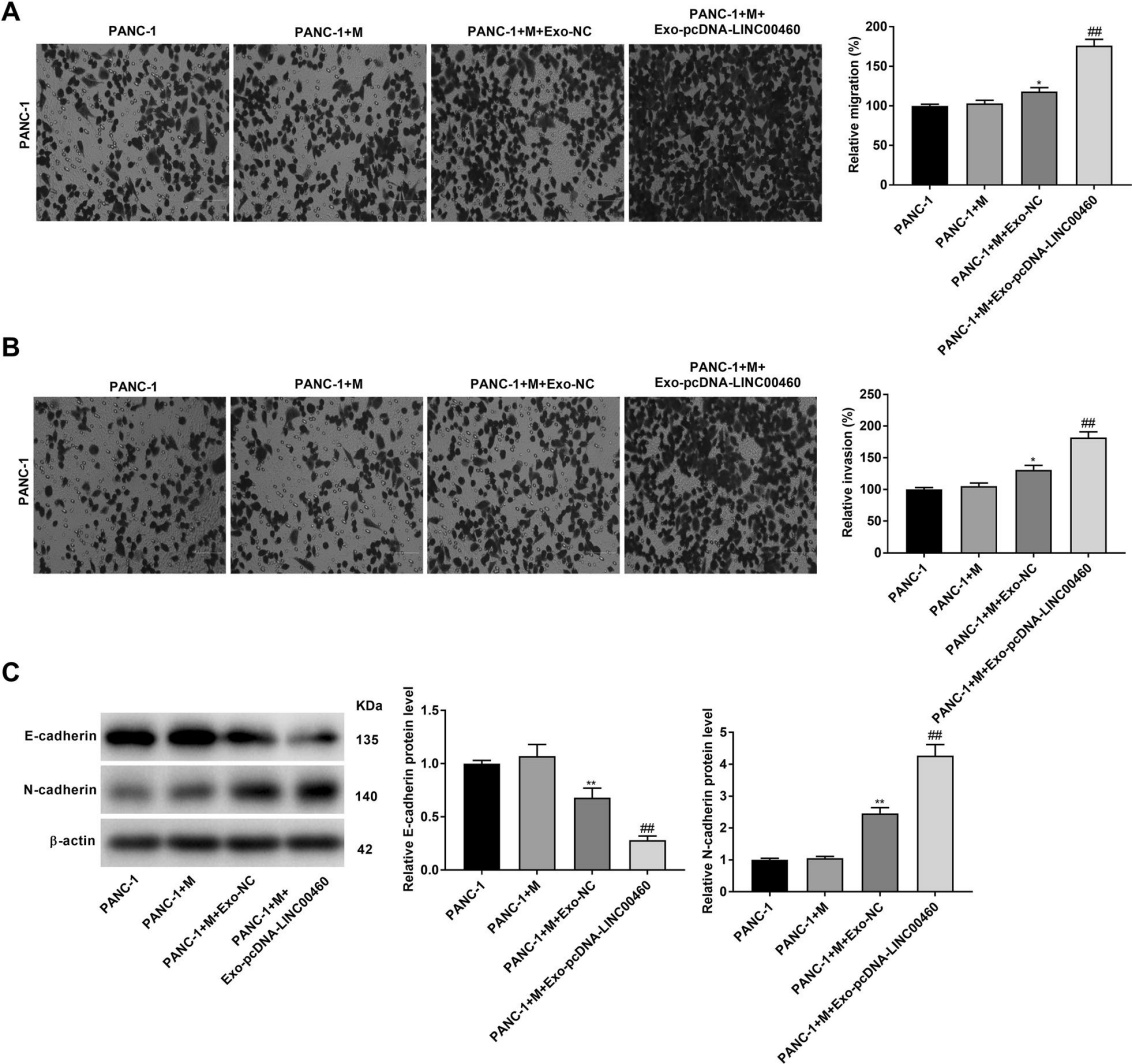
M2 macrophage polarization aggravates the migration and invasion of PANC-1 cells. **A** The migration ability of PANC-1 cells in a co-culture model was measured by transwell assay. **B** The invasion ability of PC cells in a co-culture model was measured by transwell assay. **C** The protein levels of E-cadherin and N-cadherin were determined by western blot assay. *P < 0.05, **P < 0.01 *vs*. the PANC-1 cells group; ^##^*P* < 0.01 *vs*. the PANC-1 + M + Exo-NC group

The effects of M2 macrophage polarization on the migration and invasion of PANC-1 cells was investigated using a co-culture model. As illustrated in Fig. 9A, B, we found unpolarized macrophages had no effect on cell migration and invasion. In comparison to the PANC-1 group, the abilities of migration and invasion in PANC-1 cells were both promoted in the PANC-1 + M + Exo-NC group (*P* < 0.05), whereas these situations were further facilitated in the PANC-1 + M + Exo-pcDNA-LINC00460 group (Fig. 9A, B, P < 0.01). The results indicated that exosomal LINC00460 mediated M2 macrophage polarization accelerated the metastasis of PANC-1 cells. Western blot assay demonstrated the level of pro-metastasizing protein N-cadherin in PANC-1 cells was also elevated by M2 macrophage polarization, whilst E-cadherin protein level was inhibited by M2 macrophage polarization (Fig. 9C, P < 0.01).

## Discussion

PC leads to the extremely poor prognosis (with 5-year survival rate less than 10%) due to the limitation of biomarkers for early diagnosis and potential potent therapeutic targets [52]. LncRNAs have been confirmed to function as regulators in PC progression [53, 54]. For example, Zhou et al. have uncovered that LINC00473 is highly expressed both in PC tissues and cells and its up-regulation is closely related to tumor stage and distant metastasis [53]. Wang et al. have disclosed that CRNDE expression is dramatically up-regulated in PC tissues and high expression of CRNDE is strongly associated with tumor stage and lymph node metastasis [54]. In line with these two previous studies, a visibly increased expression of LINC00460 in PC tissues and cell lines was exhibited in our study and the high expression of LINC00460 was strongly correlated with metastasis and WHO Grade, suggesting that LINC00460 may act as onco-lncRNA participation in the occurrence and development of PC.

Recently, many lncRNAs have been reported to have effects on cellular progressions of PC, including the proliferation, migration, invasion and apoptosis [53–55]. CRNDE silencing restrains the viability, migration and invasion of PC cells [54]. The ability of migration in PC cells was suppressed by ZFAS1 knockdown [55]. Another study demonstrated that inhibition of LINC00473 dampens the abilities of proliferation and metastasis of PC cells, eventually attenuating the development of PC [53]. In this study, we discovered transfection of sh-LINC00460 into SW1990 and PANC-1 cells significantly suppressed cell proliferation, migration and invasion as well as accelerated the apoptosis in vitro. Additionally, the growth of tumor xenograft was also inhibited by injection of sh-LINC00460. Similar to some of our results, a study conducted by Sun et al. has revealed that in PC cell lines (PANC-1 and SW1990 cells), transfection of si-LINC00460 has an inhibiting effect on cell proliferation [17]. However, the previous study only investigated the regulatory mechanism of LINC00460 knockdown on cell proliferation in vitro. Our results further indicated that silencing of LINC00460 not only weakened the abilities of migration and invasion, expedited cell apoptosis in vitro, but also restrained the growth of tumor xenograft in vivo. We conjectured LINC00460 silencing alleviates PC progression through inhibiting the metastasis. The results of western blot assay that sh-LNC00460 elevated the E-cadherin protein level and declined the N-cadherin protein level prove this hypothesis. The above data implied LINC00460 knockdown could retard the occurrence and development of PC effectively. Of note, increasing evidence has reported that immune evasion is essential to tumor survival and development [56]. It has been also demonstrated that in tumor microenvironment, tumor cells can recruit CD4^+^ T cells to disturb the cytotoxic functions of CD8^+^ T cells [57–59]. Furthermore, PD-L1 binds to PD-1 and further control tumor-specific T cells [19, 20]. Because of the important role of PD-1 in cancer progression, blocking PD-1 by antibody against PD-1 is considered as an efficient tool for the immunotherapy in tumors, including PC [60, 61]. Similar to the previous results, we found that the use of anti-PD-1 in mice led to the generation of smaller tumors compared to those use of IgG. Besides, the application of sh-LINC00460-1 enhanced the inhibitory effect of anti-PD-1 therapy on tumor growth in mice. We speculated silencing of LINC00460 enhances the immunogenicity of PC cells in response to anti-PD-1 therapy, and LINC00460 may be an effective target for PC immunotherapy in clinic. However, the overall efficacy of immunotherapy in clinical practice remains unpredictable due to the diversity of sociological factors, lifestyles, and metabolic disorders, and these variables should be considered before immunotherapy to achieve optimal outcomes [62].

For a long time, miR-503 has been considered to have anti-tumor effects on several types of tumors [36, 37, 63]. For instance, miR-503 is found to down-regulate in non-small cell lung cancer (NSCLC), and overexpression of miR-503 restrains the proliferation, migration and invasion of NSCLC cells [36]. MiR-503 expression is dramatically reduced in cervical cancer (CC) tissues or cells, whereas up-regulation of miR-503 contributes to dampen the growth the CC in vivo or in vitro [37–39]. Also, a study conducted by Wei et al. revealed that miR-503 is involved in suppressing the tumorigenesis of colon cancer [63]. Herein, we found a decreased expression of miR-503-5p in PC tissues and cells as well as in PAAD tissues (TCGA database). At the same time, the inhibiting effects of miR-503-5p mimics on the proliferation, migration and invasion, and the promoting effect on the apoptosis of PC cells were also exhibited in this study. Additionally, we also demonstrated that miR-503-5p was a target of LINC00460, as well as a negative correlation between LINC00460 and miR-503-5p in PC tissues. The results implied that miR-503-5p served as a downstream suppressor of LINC00460 to participate in PC progression. To verify this assumption, the feedback verification experiments were performed in PANC-1 cells. We discovered that down-regulation of miR-503-5p reversed the inhibiting effects of LINC00460 knockdown on the proliferation, migration and invasion, and the promoting effect on the apoptosis and G0/G1 phase arrest of PANC-1 cells. The results of feedback verification experiments further proved our assumption and suggested that silencing of LINC00460 alleviated the development of PC through modulating miR-503-5p.

ANLN, an onco-gene to code actin-binding protein in tumorigenesis, has been found to up-regulate in rang of human cancers, such as lung adenocarcinoma [44], nasopharyngeal carcinoma [43], cervical cancer [48], breast cancer [45, 49] and colorectal cancer [47]. In current study, ANLN was detected to highly express in PC tissues and cells. Consistent with our data, Olakowski et al. have uncovered that ANLN is overexpressed in PC [46]. The results proved the point of previous studies that ANLN might be a carcinogenic gene in PC. Simultaneously, ANLN was confirmed to be a target gene of miR-503-5p and ANLN expression was positively correlated with LINC00460. We have verified LINC00460 is involved in PC progression through regulating miR-503-5p in aforementioned conclusions. Therefore, we further speculated ANLN was also taken part in the progression of PC due to the relationships between ANLN and LINC00460/miR-503-5p. The feedback verification experiments that overexpression of ANLN reversed the inhibiting effects of LINC00460 knockdown on the proliferation, migration and invasion, and the promoting effect on the apoptosis and G0/G1 phase arrest of PANC-1 cells proved this inference. In addition, numerous lncRNAs have been reported to regulate the sensitivity of cancer cells to the cytotoxicity of tumor-reactive T cells, such as SNHG4 [64] or MALAT1 [65] in diffuse large B cell lymphoma, and KCNQ1OT1 in prostate cancer [66]. In this study, we observed that silencing of LINC00460-1 enhanced the sensitivity of PANC-1 cells to the cytotoxicity of CD8^+^ T cells cells. More importantly, the relatively high CD8^+^ T cells cytotoxicity to PANC-1 cells caused by sh-LINC00460-1 were reversed by the low expression of miR-503-5p or high expression of ANLN. These results further demonstrated that silencing of LINC00460 makes great contributions on PC immunotherapy in a cellular level. In a word, the above data suggested silencing of LINC00460 mitigated PC progression through regulating the miR-503-5p/ANLN axis.

Researchers have found that exosomes-mediated lncRNAs act as important regulators in macrophage polarization (including M1 and M2 macrophage polarization) [67–69]. LncRNA H19 mediated by exosomes elevates the levels of M1 polarization stimulators (CXCL10 and TNF-α), eventually inducing M1 macrophage polarization [67]. Whereas both exosomal lncRNA MALAT1 [68] and exosomal lncRNA BCRT1 [69] facilitate M2 macrophage polarization. Consistent with these two researches, in this study, exosomal LINC00460 from PANC-1 cells significantly increased the mRNA levels of M2 polarization markers (CD163, CD206, ARG1 and IL-10). The results indicated that exosomal LINC00460 might be a pro-M2-polarized lncRNA in PC cells. Next, we investigated the effects of M2 macrophage polarization on the migration and invasion of PANC-1 cells using a co-culture model. We found that exosomal LINC00460 induced M2 polarization expedited the progression of migration and invasion in PANC-1 cells. In line with our results, a recent study performed by Liang et al. [69] has demonstrated that M2-polarized macrophages have a promoting effect on the progression of breast cancer. Another study has been also revealed colorectal cancer cells-derived exosomal RPPH1 mediates M2 polarization, thus elevated the ability of metastasis of colorectal cancer cells [70]. We inferred M2-polarized macrophage was a kind of cells to promote tumorigenesis of PC through regulating the metastasis of PC cells. The results of western blot assay uncovered the level of pro-metastasizing protein N-cadherin was promoted by exosomal LINC00460 induced M2 macrophage polarization. Therefore, we drew a conclusion that exosomal LINC00460 from PANC-1 cells accelerated M2 macrophage polarization, whereas M2-polarized macrophage aggravated PC progression.

However, this study also exhibits some limitations. First, the down-stream mechanisms of LINC00460 in PC are not limited to the miR-503-5p/ANLN axis, and there also many other targets need to be explored. Second, the toxicological response and infiltration of CD8^+^ T cells in the model mice are not evaluated. Third, the detail regulatory mechanisms of exosomal LINC00460 involving macrophage polarization are not fully verified. Further researches on these fields are still needed.

## Conclusions

In summary, this study uncovered LINC00460 acts as an endogenous sponge of miR-503-5p and ANLN is a target gene of miR-503-5p. Silencing of LINC00460 mitigates PC progression through regulating the miR-503-5p/ANLN axis. Besides, PANC-1 cells-derived exosomal LINC00460-induced M2 macrophage polarization accelerates the migration and invasion of PANC-1 cells. The present study demonstrates that LINC00460 is essential in PC progression, pointing to it might act as a potential therapeutic target for PC.

## Supplementary Information

**Additional file 1: Figure S1. **LINC00460 knockdown inhibits the malignant characteristics of SW1990 cells. (A) The expression of LINC00460 in SW1990 cells was detected by qRT-PCR. (B) The viability (OD450) of SW1990 cells was measured by MTT assay. (C) The proliferation of SW1990 cells was determined by EdU assay (200 ×). (D) The apoptosis of SW1990 cells was analyzed by flow cytometry assay. (E) The migration ability of SW1990 cells was measured by transwell assay. (F) The invasion ability of SW1990 cells was measured by transwell assay. (G) The protein levels of E-cadherin and N-cadherin were determined by Western blot. **P < 0.01 *vs*. the sh-NC group.

**Additional file 2: Figure S2. **LINC00460 targets miR-503-5p in SW1990 cells. (A) Five miRNAs selected from databases were detected by RIP assay in SW1990 cells. **P < 0.01 *vs*. the MS2 group. (B) The luciferase activity in SW1990 cells transfected with pGL3-LINC00460 WT/pGL3-LINC00460 MUT and miR-503-5p mimics/NC was determined by DLR assay. **P < 0.01 *vs*. the miR-NC group. (C) The expression of miR-503-5p after transfection of sh-LINC00460-1/NC into SW1990 cells was detected by qRT-PCR. **P < 0.01 *vs*. the sh-NC group.

**Additional file 3: Figure S3. **Overexpression of miR-503-5p inhibits the malignant characteristics of SW1990 cells. (A) The expression of miR-503-5p after transfection of miR-503-5p mimics/NC into SW1990 cells was detected by qRT-PCR. (B) The viability (OD450) of SW1990 cells was measured by MTT assay. (C) The proliferation of SW1990 cells was determined by EdU assay (200 ×). (D) The apoptosis of SW1990 cells was analyzed by flow cytometry assay. (E) The migration ability of SW1990 cells was measured by transwell assay. (F) The invasion ability of SW1990 cells was measured by transwell assay. (G) The protein levels of E-cadherin and N-cadherin were determined by western blot assay. **P < 0.01 *vs*. the miR-NC group.

**Additional file 4: Figure S4.** MiR-503-5p targets ANLN in SW1990 cells. (A) The luciferase activity in SW1990 cells transfected with pGL3-ANLN WT/pGL3-ANLN MUT and miR-503-5p mimics/NC was determined by DLR assay. **P < 0.01 *vs*. the miR-NC group. (B) The protein level of ANLN after transfection of miR-503-5p mimics/NC into SW1990 cells was determined by western blot assay. **P < 0.01 *vs*. the miR-NC group.

## Data Availability

The datasets generated during and/or analysed during the current study are available from the corresponding author on reasonable request.

## Abbreviations

lncRNAs: Long non-coding RNAs
PC: Pancreatic cancer
PD-L1: Programmed death ligand 1
PD-1: Programmed death-1
NK: Natural killer
CTL: Cytotoxic T lymphocyte
NSCLC: Non-small-cell lung cancer
CC: Cervical cancer
ANLN: Anillin
NPC: Nasopharyngeal carcinoma
CRC: Colorectal cancer
FBS: Fetal bovine serum
IHC: Immunohistochemistry
RIP: RNA-binding protein immunoprecipitation
DLR: Dual-luciferase reporter gene
SDS-PAGE: Sodium dodecyl sulphate polyacrylamide gel electrophoresis
PVDF: Polyvinylidene fluoride
BSA: Bovine serum albumin
