# Supplementary figures and images for: Exosomal LINC00460/miR-503-5p/ANLN positive feedback loop aggravates pancreatic cancer progression through regulating T cell–mediated cytotoxicity and PD-1 checkpoint
Source: Cancer Cell Int. 2022 Dec 8;22:390. doi: 10.1186/s12935-022-02741-5 (PMC9733079; doi:10.1186/s12935-022-02741-5)

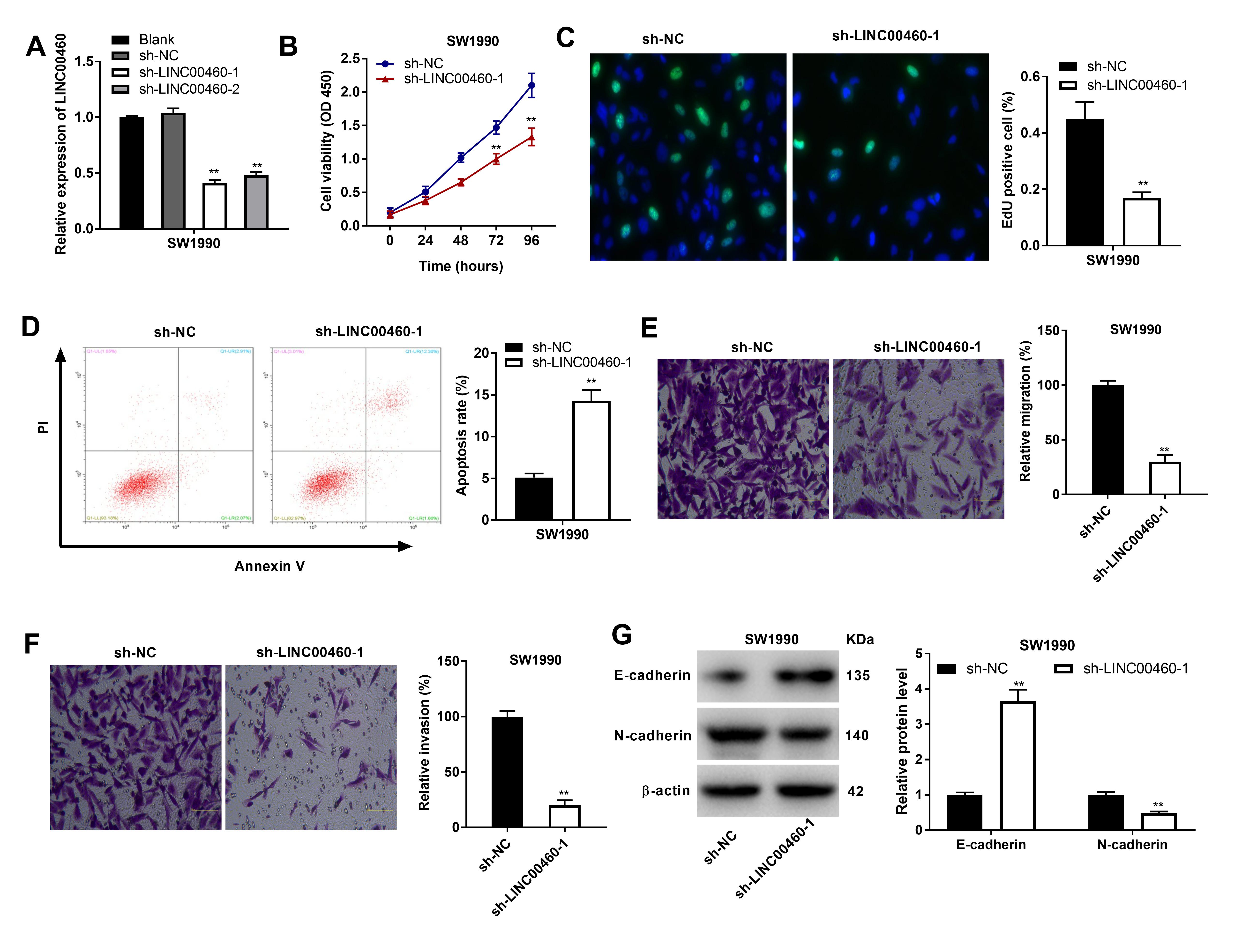

Supplement: Supplementary file 1 — Additional file 1: Figure S1. LINC00460 knockdown inhibits the malignant characteristics of SW1990 cells. (A) The expression of LINC00460 in SW1990 cells was detected by qRT-PCR. (B) The viability (OD450) of SW1990 cells was measured by MTT assay. (C) The proliferation of SW1990 cells was determined by EdU assay (200 ×). (D) The apoptosis of SW1990 cells was analyzed by flow cytometry assay. (E) The migration ability of SW1990 cells was measured by transwell assay. (F) The invasion ability of SW1990 cells was measured by transwell assay. (G) The protein levels of E-cadherin and N-cadherin were determined by Western blot. **P < 0.01 vs. the sh-NC group. [file 12935_2022_2741_MOESM1_ESM.jpg]

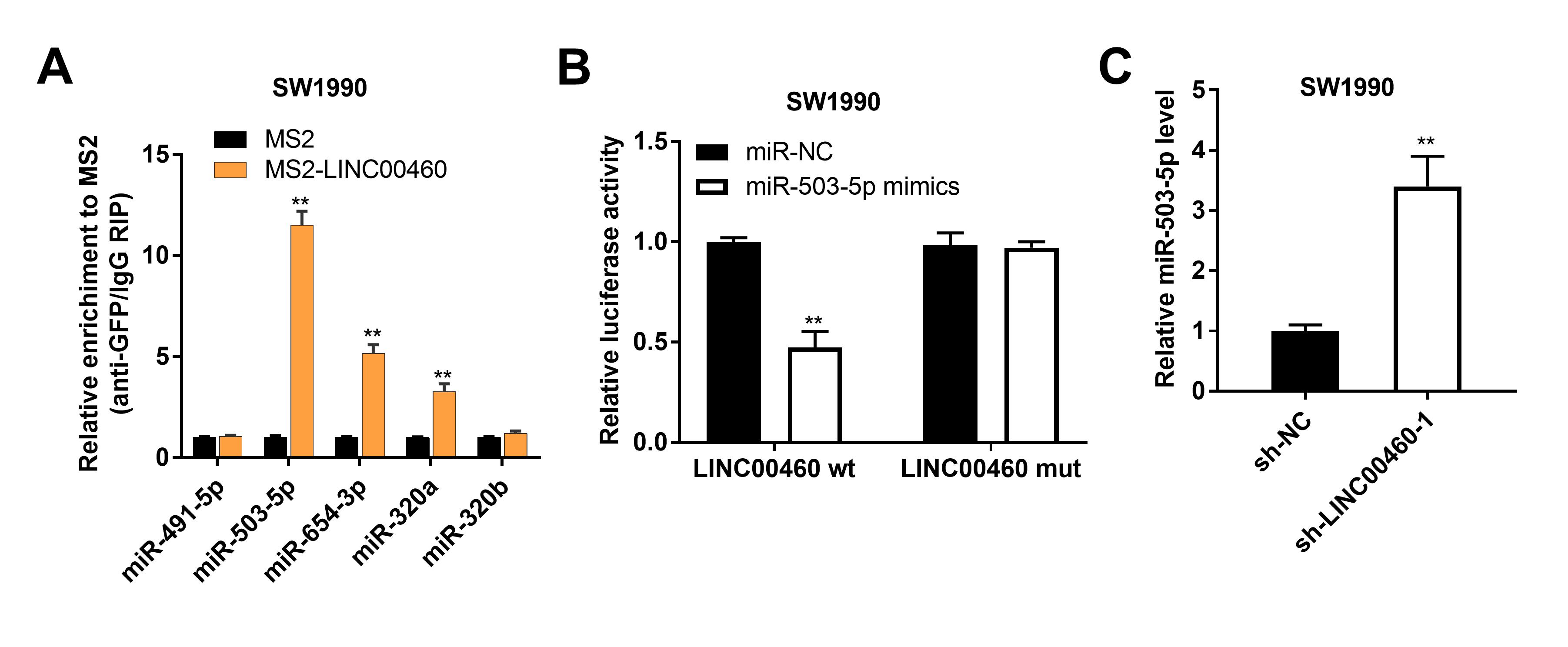

Supplement: Supplementary file 2 — Additional file 2: Figure S2. LINC00460 targets miR-503-5p in SW1990 cells. (A) Five miRNAs selected from databases were detected by RIP assay in SW1990 cells. **P < 0.01 vs. the MS2 group. (B) The luciferase activity in SW1990 cells transfected with pGL3-LINC00460 WT/pGL3-LINC00460 MUT and miR-503-5p mimics/NC was determined by DLR assay. **P < 0.01 vs. the miR-NC group. (C) The expression of miR-503-5p after transfection of sh-LINC00460-1/NC into SW1990 cells was detected by qRT-PCR. **P < 0.01 vs. the sh-NC group. [file 12935_2022_2741_MOESM2_ESM.jpg]

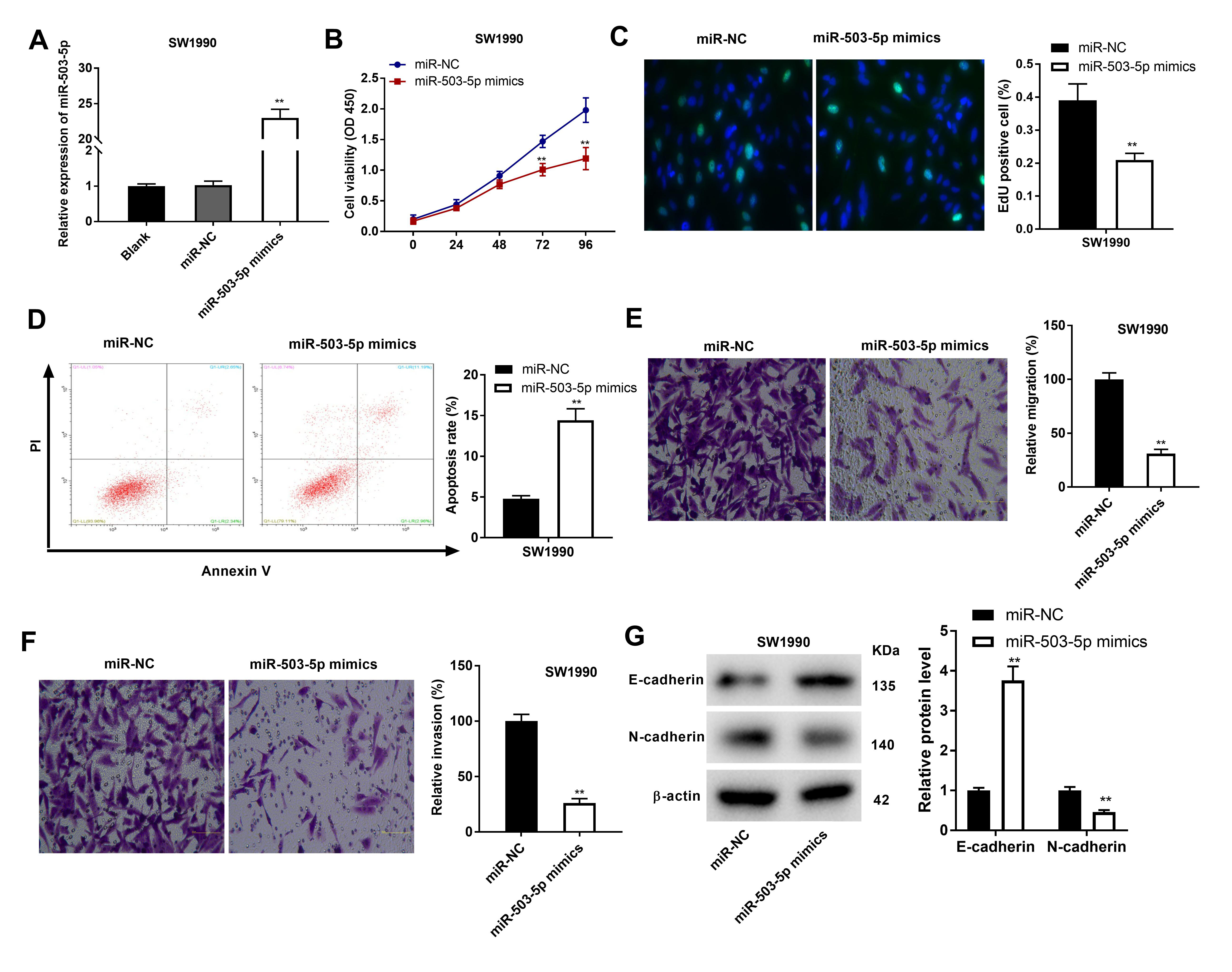

Supplement: Supplementary file 3 — Additional file 3: Figure S3. Overexpression of miR-503-5p inhibits the malignant characteristics of SW1990 cells. (A) The expression of miR-503-5p after transfection of miR-503-5p mimics/NC into SW1990 cells was detected by qRT-PCR. (B) The viability (OD450) of SW1990 cells was measured by MTT assay. (C) The proliferation of SW1990 cells was determined by EdU assay (200 ×). (D) The apoptosis of SW1990 cells was analyzed by flow cytometry assay. (E) The migration ability of SW1990 cells was measured by transwell assay. (F) The invasion ability of SW1990 cells was measured by transwell assay. (G) The protein levels of E-cadherin and N-cadherin were determined by western blot assay. **P < 0.01 vs. the miR-NC group. [file 12935_2022_2741_MOESM3_ESM.jpg]

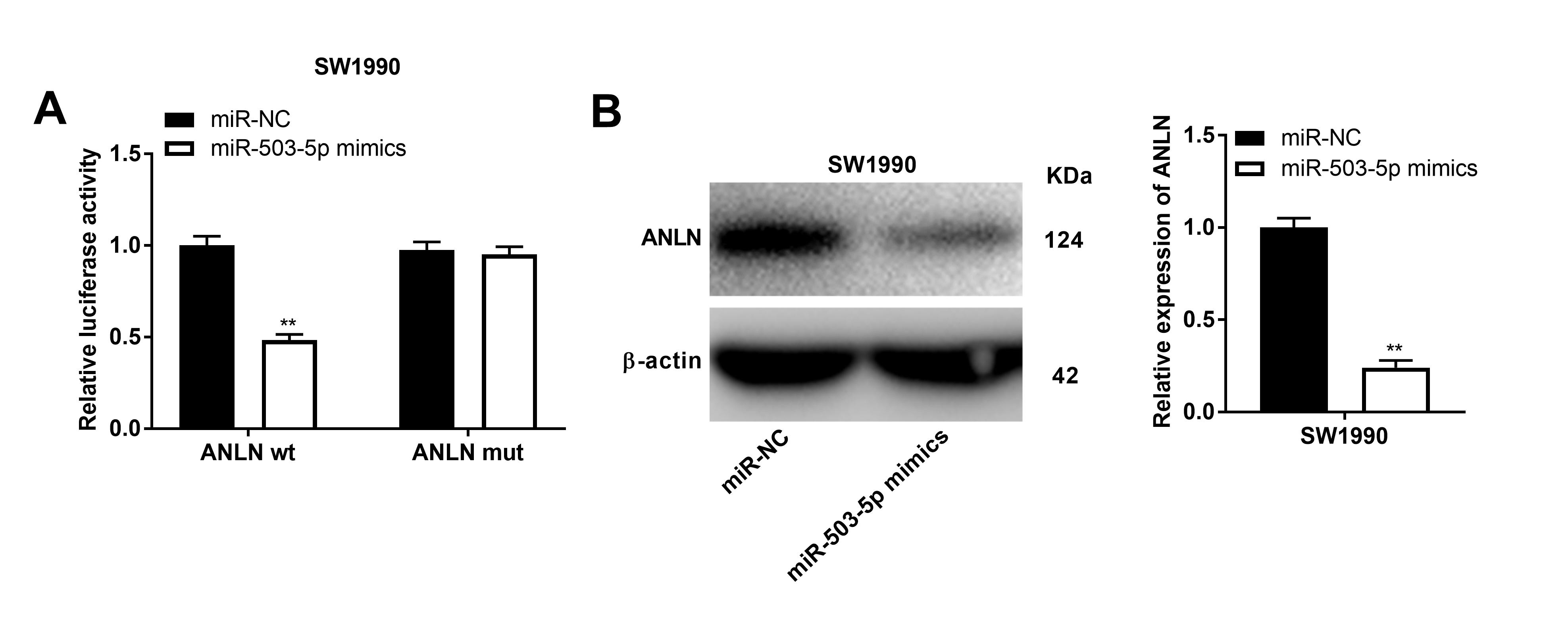

Supplement: Supplementary file 4 — Additional file 4: Figure S4. MiR-503-5p targets ANLN in SW1990 cells. (A) The luciferase activity in SW1990 cells transfected with pGL3-ANLN WT/pGL3-ANLN MUT and miR-503-5p mimics/NC was determined by DLR assay. **P < 0.01 vs. the miR-NC group. (B) The protein level of ANLN after transfection of miR-503-5p mimics/NC into SW1990 cells was determined by western blot assay. **P < 0.01 vs. the miR-NC group. [file 12935_2022_2741_MOESM4_ESM.jpg]
